# Molecular Mechanisms of Phase Separation and Amyloidosis of ALS/FTD-linked FUS and TDP-43

**DOI:** 10.14336/AD.2023.1118

**Published:** 2024-10-01

**Authors:** Jianxing Song

**Affiliations:** Department of Biological Sciences, Faculty of Science; National University of Singapore; 10 Kent Ridge Crescent, Singapore.

**Keywords:** FUS, TDP-43, Intrinsically-disordered region (IDR), protein misfolding, liquid-liquid phase separation (LLPS), Amyloidosis, Amyotrophic lateral sclerosis (ALS)

## Abstract

FUS and TDP-43, two RNA-binding proteins from the heterogeneous nuclear ribonucleoprotein family, have gained significant attention in the field of neurodegenerative diseases due to their association with amyotrophic lateral sclerosis (ALS) and frontotemporal degeneration (FTD). They possess folded domains for binding ATP and various nucleic acids including DNA and RNA, as well as substantial intrinsically disordered regions (IDRs) including prion-like domains (PLDs) and RG-/RGG-rich regions. They play vital roles in various cellular processes, including transcription, splicing, microRNA maturation, RNA stability and transport and DNA repair. In particular, they are key components for forming ribonucleoprotein granules and stress granules (SGs) through homotypic or heterotypic liquid-liquid phase separation (LLPS). Strikingly, liquid-like droplets formed by FUS and TDP-43 may undergo aging to transform into less dynamic assemblies such as hydrogels, inclusions, and amyloid fibrils, which are the pathological hallmarks of ALS and FTD. This review aims to synthesize and consolidate the biophysical knowledge of the sequences, structures, stability, dynamics, and inter-domain interactions of FUS and TDP-43 domains, so as to shed light on the molecular mechanisms underlying their liquid-liquid phase separation (LLPS) and amyloidosis. The review further delves into the mechanisms through which ALS-causing mutants of the well-folded hPFN1 disrupt the dynamics of LLPS of FUS prion-like domain, providing key insights into a potential mechanism for misfolding/aggregation-prone proteins to cause neurodegenerative diseases and aging by gain of functions. With better understanding of different biophysical aspects of FUS and TDP-43, the ultimate goal is to develop drugs targeting LLPS and amyloidosis, which could mediate protein homeostasis within cells and lead to new treatments for currently intractable diseases, particularly neurodegenerative diseases such as ALS, FTD and aging. However, the study of membrane-less organelles and condensates is still in its infancy and therefore the review also highlights key questions that require future investigation.

## 1. Introduction

Liquid-liquid phase separation (LLPS) is now recognized as a common principle governing the assembly of various membraneless organelles and cellular condensates. These membraneless organelles include nucleoli, Cajal bodies, nuclear speckles, paraspeckles, histone-locus bodies, nuclear gems, and promyelocytic leukemia (PML) bodies within the nucleus, along with P-bodies, stress granules (SGs), and germ granules in the cytoplasm [1-6]. Previously, extensive research focused on the phase separation of well-folded proteins, such as lysozyme, revealing its occurrence only at high concentrations (>mM) [6-8]. In contrast, proteins that play a vital role in the formation of membraneless organelles possess large intrinsically-disordered regions (IDRs), enabling phase separation at considerably lower concentrations (~μM). Fundamentally, the multivalency of binding sites within an IDR-rich protein facilitates simultaneous interactions with multiple copies of itself (homotypic phase separation) or other biomolecules (heterotypic phase separation). Consequently, LLPS occurs, resulting in the division of a well-mixed homogeneous solution into two coexisting phases: a dense phase and a dilute phase [1-16]. LLPS is a dynamic and reversible phenomenon, characterized by the continuous exchange of molecules between the dense and diluted phases. Despite this exchange, the concentration within the dense phase significantly exceeds that of the dilute phase, often by more than 50 times [15]. This may lead to the aging of dynamic droplets, transitioning from a liquid-like state to a more solid-like state, ultimately resulting in the formation of cytoplasmic inclusions or amyloid fibrils, which are characteristic of various diseases. In the past decade, exponential studies have not only revealed the crucial role of LLPS in cellular functions but also shed light on its involvement in disease processes.

Proteinopathies, ranging from well-structured cross-β amyloid fibrils and less organized amorphous inclusions, represent a universal hallmark for an increasing spectrum of human diseases. These diseases include all neurodegenerative disorders such as amyotrophic lateral sclerosis (ALS) and frontotemporal dementia (FTD) [16-23], and even extend to aging processes, affecting organisms down to *E. coli* cells [24]. Despite extensive research, the mechanisms by which misfolding-/aggregation-prone proteins gain toxicity and initiate disease and aging processes remain a great mystery. ALS is the most prevalent motor neuron disease, which was initially described in 1869, but its underlying mechanism largely remains enigmatic. On the other hand, FTD is a neurodegenerative disorder characterized by progressive deterioration of behavior, language, and executive functions. It is the second most common cause of early-onset dementia after Alzheimer's disease. ALS and FTD not only have a significant overlap in the aggregation-prone proteins, but also share overlapping clinical and pathological features, suggesting a close relationship and potential common mechanisms between the two disorders. Similar to other neurodegenerative diseases, ALS/FTD are marked by proteinopathies both *in vivo* and *in vitro* [16-22,25]. ALS-associated proteins can be classified into two main groups. The first group consists of proteins like human Cu/Zn superoxide dismutase (hSOD1) (26-28) and profilin-1 (hPFN1) [29-32], wherein the wild-type proteins possess well-folded functional structures and high solubility. However, their ALS-causing mutants are prone to misfolding and aggregation, acquiring additional cytotoxicity [26-32]. The second group including FUS and TDP-43, which are also associated with FTD, is inherently aggregation-prone and toxic even in their wild-type forms [16,33-36].

Recent discoveries have unveiled that proteins prone to misfolding/aggregation share a common tendency to accumulate and aggregate within stress granules (SGs) [4,37-40]. SGs are membrane-less organelles which are formed through LLPS of RNA-binding proteins (RBPs) containing prion-like domains like FUS and TDP-43 [33-40]. Intriguingly, the accumulation of misfolding/aggregation-prone proteins disrupts the dynamics of SGs, offering a potential mechanism by which these proteins acquire toxicity, triggering diseases and aging. SGs have also been proposed to be a novel protein quality control (PQC) machinery that non-classically manages misfolded/aggregation-prone proteins, making them a central target in various diseases, including ALS and FTD [4,37-39]. Notably, the ALS-causing C71G mutant of hPFN1 has been observed to induce seed-dependent co-aggregation with FUS/TDP-43, thus manifesting a prion-like propagation [41,42].

FUS and TDP-43 are members of the RNA-binding protein (RBP) family, which exhibit a modular architecture similar to heterogeneous nuclear ribonucleoproteins (hnRNPs). Notably, FUS and TDP-43 possess a distinctive domain structure marked by the presence of significant intrinsically disordered regions (IDRs), including prion-like domains (PLDs), which are prone to aggregation, posing toxicity to cells [43,44]. Additionally, both proteins feature structured domains responsible for binding various nucleic acids, such as DNA and RNA. The folded domains comprise RNA-recognition motifs (RRMs) in both FUS and TDP-43, Zinc finger (ZnF) in FUS, and the N-terminal domain (NTD) in TDP-43. They perform vital roles in various cellular processes, such as transcription, splicing, microRNA maturation, RNA stability and transport and DNA repair, as well as stress granule (SG) formation.

Unlike most other RBPs, which are typically confined to the nucleus or cytoplasm without shuttling between the two, TDP-43 and FUS exhibit the ability to shuttle between the nucleus and cytoplasm in response to different stimuli. While primarily nuclear in glial cells and neurons, both FUS and TDP-43 can translocate to the cytoplasm during environmental stresses, participating in stress granules (SGs) formed through homotypic and heterotypic phase separation driven by nucleic acid interactions. Interestingly, FUS and TDP-43, along with their mutants, can undergo mislocalization and aggregation, implicated in various neurodegenerative diseases beyond ALS and FTD. For example, TDP-43 and FUS are extensively associated with Alzheimer's disease (AD), Parkinson's disease (PD), and Huntington's disease (HD) [16,25,33,36,43-50].

In this context, decoding molecular mechanisms of LLPS and amyloid formation of TDP-43 and FUS is pivotal for unraveling the mystery of the associated neurodegenerative diseases and also bears critical clinical implications. This includes developing new diagnostic tools and prognostic markers. For instance, creating imaging techniques to visualize LLPS and protein aggregates in the brain in vivo, as well as identifying blood-based biomarkers, could diagnose neurodegenerative diseases earlier and more accurately [45,51-54]. Ultimately, this knowledge will establish therapeutic targets for developing drugs that target proteins involved in LLPS and protein aggregation, as well as drugs that modulate the cell environment to promote normal LLPS droplet formation and prevent pathological protein aggregate formation [55,56]. Understanding LLPS and protein aggregation in neurodegenerative diseases is still in its early stages, yet it is a rapidly growing research field with the potential to transform disease diagnosis, prognosis, and treatment.

The literature reviewed here extensively employs three major biophysical methods: X-ray crystallography, cryo-electron microscopy (Cryo-EM), and NMR spectroscopy. X-ray crystallography is a classic technique for determining the atomic structure of proteins, as well as amyloid fibrils. However, to determine the crystal structure of a protein or amyloid fibril, researchers first need to grow high-quality single crystals of the protein or amyloid fibril. This can be an extremely challenging process, as it requires the protein or amyloid fibril to be in a very pure and ordered state. On the other hand, cryo-electron microscopy (Cryo-EM) determined three-dimensional structures of biological molecules by freezing a sample of the protein or amyloid fibril in a thin layer of water, which is then bombarded with electrons. The electrons interact with the protein or amyloid fibril, creating images that can be analyzed to determine the structure of the molecule. Cryo-EM has several advantages over X-ray crystallography: crystals are not required, and proteins can be studied in solution, which is more representative of their natural environment. Cryo-EM can be used to study large proteins, intricate protein complexes, and challenging structures like membrane proteins and amyloid fibrils that are hard to crystallize. In this context, despite Cryo-EM being a less well-established method with lower resolution and more complex data analysis, it represents a rising technique used to determine the structures of amyloid fibrils associated with various diseases, including Amyotrophic lateral sclerosis, Alzheimer's disease, Parkinson's disease, and Huntington's disease [57-59].

X-ray crystallography is not well-suited for studying LLPS, as it necessitates the formation of a single crystal. Additionally, while Cryo-EM can capture the dynamic behavior of the system in real time, to obtain high-resolution insights into LLPS still remains challenging for Cryo-EM at present. On the other hand, NMR spectroscopy is a powerful tool for directly studying the structure, dynamics, and interactions of proteins in solution, which include intrinsically disordered proteins (IDPs) as well as their phase separation [60-62]. Briefly, Several NMR parameters may be particularly suitable for characterizing IDPs and their phase separation. These include chemical shifts, nuclear Overhauser effects (NOEs) and relaxation rates. Chemical shifts provide information about the secondary structures of proteins, the local chemical environment of each atom and its interactions. NOEs provide information about the distance between different atoms in the protein. Relaxation rates provide information about the dynamics of the protein. By measuring these parameters, scientists can gain high-resolution knowledge for the structure, dynamics, and interactions of IDPs and their phase separation.

Currently, there are numerous excellent reviews available on FUS and TDP-43. Therefore, this review aims to offer a cohesive and comprehensive overview of the biophysical understanding of the structures, stability, dynamics, and inter-domain interactions of FUS and TDP-43 domains, as well as their mechanisms underlying LLPS and amyloidosis, with a particular focus on how these processes are modulated by ATP and nucleic acids. Additionally, this review delves into the biophysical mechanism through which ALS-causing mutants of the well-folded hPFN1 disrupt the dynamics of LLPS of FUS NTD. By integrating these diverse pieces of information, the review strives to offer an up-to-date biophysical understanding of FUS and TDP-43, shedding light on their crucial roles in disease pathogenesis. Finally, the review also raises open questions in this rapidly evolving field.

## 2. Fused in sarcoma (FUS)

### 2.1. Amino acid composition, structure and stability of FUS domains

FUS, also known as Fused in sarcoma (FUS) or Translocated in Sarcoma (TLS), belongs to the FUS/TLS, EWS, and TAF15 (FET) protein family. It is a multi-functional heterogeneous nuclear ribonucleoprotein (hnRNP) that plays a pivotal role in regulating various cellular processes through its binding to large array of nucleic acids, such as DNA and RNA to control transcription, RNA processing, cytoplasmic fates of mRNAs, and DNA damage responses [63-65]. FUS also acts as a component of cellular granules, specifically stress granules (SGs), which are formed with mRNA in response to environmental stresses. However, the mislocalization and inclusions of FUS and its mutant forms in the cytoplasm have been associated with various neurodegenerative disorders, particularly ALS and FTD. The abnormalities disrupt normal cellular processes, including RNA metabolism, leading to impaired neuronal function and eventual cell death [43,44,66-69]. Furthermore, FUS abnormalities have also been implicated in other diseases such as essential tremor, myotonic dystrophy, and certain types of cancer [70].

**Figure 1.**
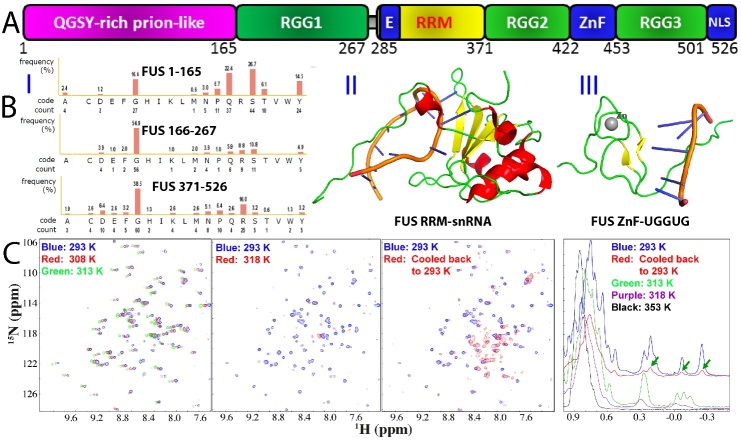
Domain organization, sequence and structure of FUS. (A) Domain organization of 527-residue human FUS protein. (B) (I) Amino acid compositions of intrinsically-disordered NTD consisting of the prion-like domain (1-165) and RGG1 (166-267), as well as CTD (371-526). Three-dimensional structures of RRM in complex with snRNA (II), and Zinc-finger (ZnF) in complex with RNA (UGGUG) (III). (C) NMR HSQC and 1D spectra of FUS RRM domain at different temperatures. Green arrows are used to indicate several very up-field peaks characteristic of the folded RRM domain.

The 526-residue FUS consists of three main domains (Fig 1A): the N-terminal domain (NTD) comprising the QGSY-rich prion-like domain (PLD) from 1 to 165 with high sequence identity to the N-terminal yeast prion domain of Sup35 [71-73], an RG/RGG-rich region 1 (RGG1) spanning 166 to 267, a middle RNA-recognition motif (RRM) domain from 282 to 371, and the C-terminal domain (CTD) from 371 to 526, which includes RGG2, a zinc finger (ZnF), and RGG3. Notably, more than 70% of the FUS sequence comprises IDRs, characterized by the absence of any bulky hydrophobic residues Leu, Ile, and Val. Intriguingly, these IDRs can be classified into two distinct types: the polar/aromatic residue-rich PLD (1-165), which contains 24 Tyr but lacks any Arg/Lys, and the RG-/RGG-rich regions from 166 to 267 and the CTD (371-526), which are abundant in Gly and Arg/Lys (I of Fig. 1B).

FUS consists of two folded domains: an RNA-recognition motif (RRM) domain (II of Fig. 1B), and a zinc finger (ZnF) within the C-terminal domain (CTD) (III of Fig. 1B). These domains have the ability to bind nucleic acids in both sequence-specific and sequence-independent manners [74-78]. The RRM domain, which carries the conserved RNP1 and RNP2 sequence stretches, is one of the most abundant domains in eukaryotes. Most heterogeneous nuclear ribonucleo-proteins (hnRNP) contain one or several RRM domains that mediate the direct interaction with nucleic acids. Notably, despite significant sequence variation from other RRMs, the NMR structures of FUS RRM have been found to adopt a similar overall fold to other RRMs in its free state [75] or in complex with RNA [76,77]. This fold is characterized by a four-stranded β-sheet and two perpendicular α-helices. However, the FUS RRM does possess distinct features, including an elongated and positively charged "KK" loop. Moreover, its nucleic acid binding pocket is structurally distorted, with the absence of several key aromatic residues. Interestingly, the "KK" loop plays a key role in binding RNA and DNA, and the binding affinity falls within the micromolar range (II of Fig. 1B).

The FUS ZnF spanning residues 418-454 belongs to the C4 subfamily of the zinc finger superfamily [76,79]. Interestingly, its folding is primarily dependent on zinc coordination, as it lacks large hydrophobic residues such as Val, Ile, or Leu. Consequently, in the absence of nucleic acid binding, the FUS ZnF exhibits a relatively loose tertiary packing, despite showing a well-dispersed HSQC spectrum [79]. This behavior is reminiscent of a previous discovery where a small protein consisting of only 37 residues, constrained by disulfide bridges, maintained well-dispersed NMR spectra even when its tightly packed sidechains were disrupted by acid unfolding [80-82]. Nevertheless, upon forming a complex with the UGGUG RNA fragment, the FUS ZnF adopted a well-structured conformation, and its NMR structure was determined [76]. The structure of the FUS ZnF includes zinc ribbon-like domains characterized by two crossed β-hairpins, with a zinc atom coordinated by four cysteines (III of Fig. 1B).

Protein misfolding, aggregation, and amyloidosis are strongly dependent on both thermodynamic and kinetic stability [78,83,84]. Biophysical investigations utilizing circular dichroism (CD) and fluorescence spectroscopy have revealed that the FUS RRM exhibits relatively low thermodynamic stability, characterized by a melting temperature of only 52 °C. Interestingly, despite the absence of any cysteine (Cys) residues in the FUS RRM, unfolding of this domain is irreversible. As illustrated in Figure 1C, FUS RRM exhibits a well-folded conformation at 20 °C, as evidenced by a well-dispersed HSQC spectrum. However, upon raising the temperature to 45 °C, significant denaturation occurs, leading to the disappearance of most HSQC peaks. Moreover, at 80 °C, the RRM domain undergoes extensive denaturation, causing all up-field NMR peaks to completely vanish. Interestingly, upon cooling the sample back to 20 °C, the majority of HSQC peaks remain undetectable, and 1D peaks do not fully align with those of the initial RRM sample at 20 °C. This observation clearly demonstrates the irreversibility of the unfolding process, suggesting that the unfolded FUS RRM may undergo irreversible self-association, thereby hindering its refolding into the original structure [78].

### 2.2. LLPS of FUS domains

FUS is primarily localized in the nucleus in most cell types. It is known, however, to shuttle between the nucleus and the cytoplasm, participating in various cellular processes. Within the nucleus, FUS can be found associated with chromatin, contributing to transcriptional regulation and RNA processing. It is also a key component of nuclear bodies, including the Cajal bodies and the nucleolus, where it plays roles in RNA metabolism and ribosome biogenesis. In the cytoplasm, FUS is abundantly present in SGs, which harbor mRNA and various RNA-binding proteins for mRNA storage and translational repression. FUS has been extensively demonstrated to establish high-order functional assemblies with nucleic acids through the process of phase separation [85-87].

**Figure 2.**
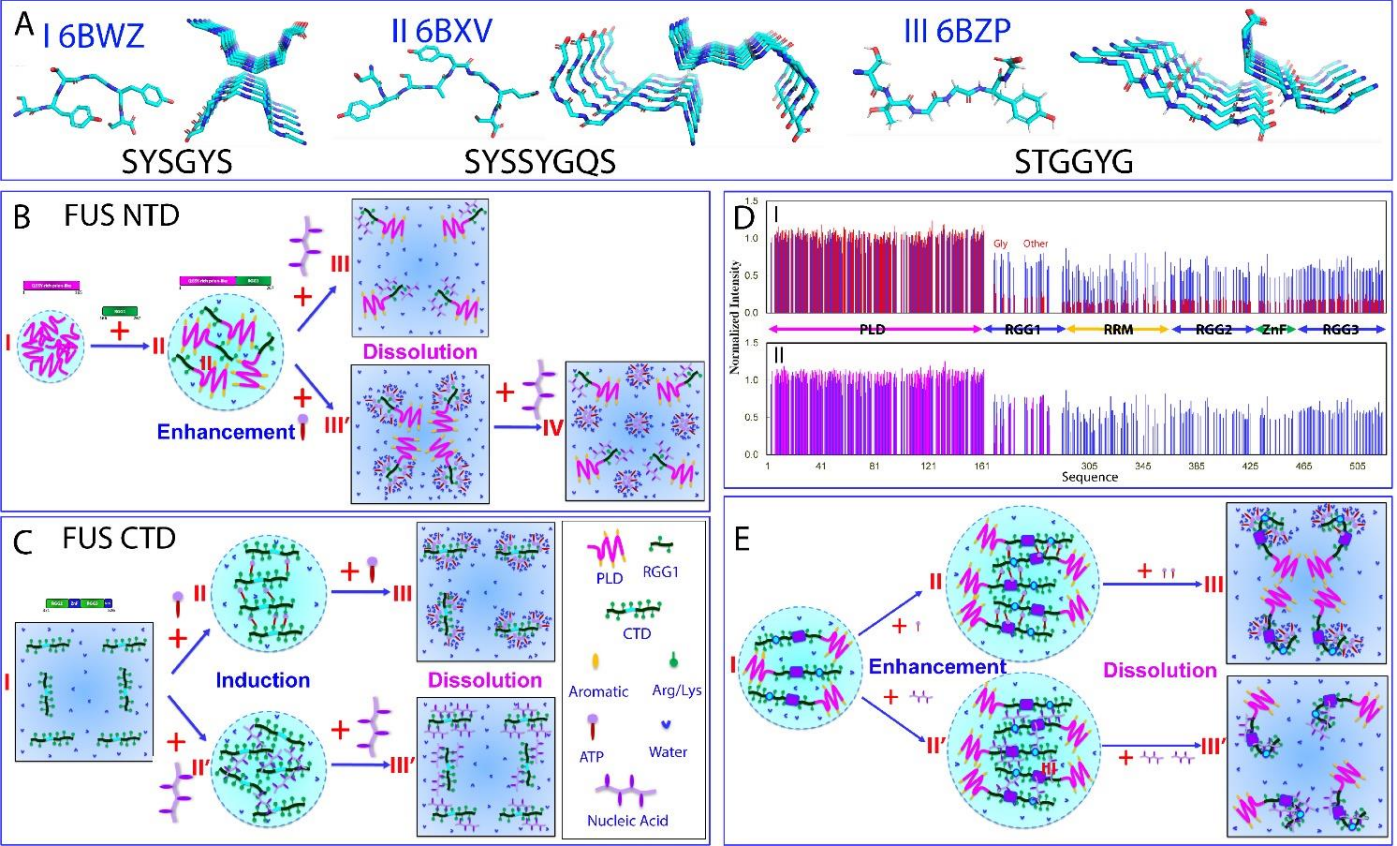
LLPS of FUS and its modulation by ATP and ssDNA. (A) Structures of three low-complexity aromatic-rich kinked segments (LARKS) of FUS PLD. Speculative models to illustrate ATP and nucleic acids to interplay to modulate LLPS of FUS NTD (B); and FUS CTD (C). (D) (I) Normalized HSQC peak intensity of the ^15^N-labeled FUS in the presence of TssDNA at molar ratios of 1: 0.1 (blue) and 1:0.5 (red) as divided by that of FUS in the free state. (II) Normalized HSQC peak intensity of the ^15^N-labeled FUS in the presence of TssDNA at molar ratios of 1: 0.1 (blue) and 1:10 (purple) as divided by that of FUS in the free state. (E) A speculative model to rationalize the specific binding of ATP and ssDNA to Arg/Lys residues as well as RRM and ZnF of FUS to enhance LLPS at low concentrations but to dissolve at high concentrations.

Systematic dissection studies in vitro have revealed that in the absence of nucleic acids, only FUS NTD possesses the ability to phase separate autonomously. Conversely, its RRM and CTD lack this inherent capacity [78, 79]. FUS NTD (1-267) contains PLD (1-165), which is rich in serine, tyrosine, glycine, and glutamine (QGSY-rich) residues, but lacks Arg/Lys and large hydrophobic residues (I of Fig. 2A). This domain has been shown to play a crucial role in facilitating self-assembly into liquid-like granules, which could mature into hydrogels and solid aggregates [86,87]. In hydrogels, FUS NTD became non-covalently polymerized, forming morphologically homogeneous amyloid-like fibrils. Analysis using X-ray fiber diffraction and electron microscopy decoded the presence of cross-β structures in these fibrils, resembling those found in pathogenic amyloid and prion fibrils. However, unlike pathogenic fibrils, FUS NTD fibrils exhibit lability and reversibility, which can be easily disassembled through dilution, temperature changes, or mild detergent treatment [86].

FUS PLD consists of 24 tyrosine residues arranged in repetitive patterns, usually with a consensus sequence of [S/G]Y [S/G], followed by one to three glutamine or proline residues. Mutations of Tyr residues decreased the ability to phase separate, depending on the number rather than the position of these substitutions. Substituting all Tyr residues with nonaromatic residues nearly eradicated this capability, highlighting the importance of π-π interactions involving aromatic residues [88]. Residue-specific NMR studies have revealed that FUS PLD adopts solution conformations lacking any classic secondary structures, both in its free state and within phase-separated droplets. Moreover, the stabilization of LLPS of FUS PLD is attributed to a combination of hydrogen bonding, π/sp2 interactions, and hydrophobic interactions. Notably, in addition to tyrosine residues, glutamine residues also engage in interactions that drive LLPS of FUS PLD [89, 90]. Indeed, the atomic structures have been determined for three FUS NTD segments: _37_SYSGYS_42_, _54_SYSSYGQS_61_, and _77_STGGYG_82_ (I-III of Fig. 2A). These structures exhibit a shared characteristic: the arrangement of segments into kinked β-sheets that form protofilaments. However, unlike steric zippers found in amyloid fibrils, the interaction between the kinked sheets is weak due to the engagement of polar atoms and aromatic side chains, thus termed as low-complexity aromatic-rich kinked segments (LARKS) [91].

On the other hand, *in vitro* and *in vivo* studies have also demonstrated the indispensability of both PLD and RGG regions in the phase separation of full-length FUS [11,78,79,92-94]. Indeed, FUS PLD phase separated only at high concentrations or in the presence of dextran, a molecular crowding agent [11, 89] (I of Fig. 2B). Intriguingly, NMR investigations uncovered that FUS NTD with the inclusion of RGG1, which lacks autonomous phase separation capability, could phase separate at remarkably low concentrations (~1 μM) to form large droplets [78] (II of Fig. 2B). Furthermore, it was observed that FUS PLD also underwent phase separation at low concentrations when the isolated RGG3 region was added [95]. These findings strongly support the notion that in the full-length FUS, π-π or/and π-cation interactions involving aromatic amino acids and Arg/Lys residues significantly contribute to driving LLPS of FUS [78-79,92-95].

From a physiological perspective, FUS functions within cellular environments that contain diverse nucleic acids including RNA and DNA [96]. Interestingly, ATP also exists in all living cells with high concentrations, ranging from 1 to 12 mM depending on the cell type, despite ATP-dependent proteins and enzymes requiring only micromolar concentrations of ATP for their functioning [97-101]. Intriguingly, *in vitro* investigations have unveiled that ATP >6 mM serves as a biological hydrotrope, effectively dissolving LLPS of RNA-binding proteins (RBPs) including FUS [98, 99]. Furthermore, ATP exhibits a dual role in the LLPS of FUS by acting as a bivalent binder: it induces phase separation at low concentrations but dissolves it at high concentrations [79, 101]. Consequently, a pivotal question of fundamental and therapeutic relevance arises regarding the effects and mechanisms of ATP and nucleic acids to modulate LLPS of FUS.

To address this question, a systematic study aimed to elucidate the molecular interactions driving LLPS of FUS and its dissected domains as well as its modulation by ATP, RNA, and ssDNA of specific and non-specific sequences [79]. The FUS domains analyzed include PLD (1-165), NTD (1-267) and CTD (371-526). The RNA sequence used was UAGUUUGGUGAU, while the ssDNAs employed were telomeric ssDNA (TssDNA) with the sequence (TTAGGG)_4_, which forms a G-quadruplex tertiary structure, and (TTTTTT)_4_ (T24) without any secondary or tertiary structures. Intriguingly, for FUS PLD, at concentrations where it lacks the ability to undergo phase separation, ATP, RNA, and ssDNA were also unable to induce its phase separation. Furthermore, NMR studies have demonstrated that these molecules do not exhibit significant interactions with FUS PLD. In contrast, for FUS NTD (1-267) which has the inherent phase separation at very low concentrations, ATP, RNA, and two ssDNA molecules have been found to exert a monotonic dissolution of its LLPS. The extent of dissolution varies depending on the length of the nucleic acids. Subsequent NMR studies elucidated the underlying mechanism, revealing that ATP and the nucleic acids achieve this dissolution by binding to the same set of residues within the RGG1 region. As depicted in II of Figure 2B, LLPS of FUS NTD appears to be primarily driven by π-π or/and π-cation interactions between aromatic amino acids in PLD and Arg/Lys residues in RGG1. In this context, nucleic acids with multiple base aromatic rings can also establish π-π or/and π-cation interactions with the Arg/Lys residues in RGG1, with the binding affinity depending on the binding multivalency [79,102]. Consequently, the base aromatic rings of the nucleic acids competitively displace the aromatic rings of PLD aromatic residues from binding to Arg/Lys residues in RGG1, leading to the dissolution of LLPS of FUS NTD (III of Fig. 2B).

By a similar mechanism, ATP can also utilize the adenine aromatic ring to establish π-π or/and π-cation interactions with the Arg/Lys residues in RGG1, albeit with much lower affinity compared to nucleic acids. Under this context, only when the concentration of ATP is sufficiently high, the adenine aromatic ring of ATP clusters around the Arg/Lys residues in RGG1, competitively disrupting their interactions with aromatic residues within PLD and causing the dissolution of FUS NTD's LLPS. However, unlike nucleic acids, the triphosphate group of ATP appears to strongly interact with water molecules. As a result, the NTD molecules bound with ATP self-assemble into large and dynamic oligomers, involving the aromatic/hydrophobic PLD residues, despite the absence of strong binding with ATP (III' of Fig. 2B). Due to the size and dynamics of the ATP-NTD oligomers, most NMR signals become too broad to detect [79,101]. However, upon introducing ssDNA molecules, which have a much higher affinity for Arg/Lys residues, the ATP molecules are competitively displaced from being clustered around these residues, leading to the disassembly of ATP-NTD oligomers (IV of Fig. 2B). Consequently, the NTD molecules were shifted to be bound with ssDNA, and most NMR HSQC peaks become detectable [79,101].

On the other hand, FUS CTD (371-526) does not exhibit the capacity in phase separation under various conditions (I of Fig. 2C). However, the introduction of ATP, RNA, TssDNA, or T24 all induces its phase separation, followed by subsequent dissolution. Briefly, at low concentrations, ATP acts as a bivalent binder, utilizing the aromatic ring of its adenine group to bind to the side chains of Arg/Lys and its triphosphate chain to bind Arg/Lys. This binding leads to the formation of large and dynamic ATP-CTD complexes, manifesting as liquid droplets (II of Fig. 2C). However, at high concentrations, the excess binding of ATP disrupts these complexes, resulting in the dissolution of the droplets (III of Fig. 2C). Interestingly, unlike FUS NTD, FUS CTD lacks aromatic-residue-rich regions that can further self-assemble into large oligomers when over-bound with ATP (III' of Fig. 2B). Therefore, HSQC peaks of ATP-bound FUS CTD molecules remain detectable. Similarly, nucleic acids also bind to Arg/Lys residues to induce and dissolve phase separation, similar to ATP, but with a higher affinity due to their ability to establish multivalent binding to FUS CTD (II’ and III’ of Fig. 2C).

Indeed, detailed analysis of NMR data on CTD revealed remarkably similar patterns of chemical shift differences (CSD) induced by ATP, TssDNA, and T24 (79). Notably, a significant number of residues, including 25 Arg and 4 Lys residues, exhibited noticeable shifts in their HSQC peaks. This observation strongly suggests specific binding of ATP and ssDNA to these Arg/Lys residues [79]. Interestingly, the binding of ATP and ssDNA to Arg/Lys residues is not highly dependent on the specific sequence hosting these Arg/Lys residues, as long as the region remains disordered. These findings provide compelling evidence that Arg/Lys residues are crucial for ATP and nucleic acids to induce and dissolve phase separation in FUS CTD [79,100].

For the full-length FUS, as exemplified by TssDNA, its addition at low molar ratios led to no significant shift of HSQC peaks but only changes in peak intensity. However, upon addition of TssDNA at a ratio of 1:1, under which LLPS was significantly enhanced, all HSQC peaks disappeared. Unexpectedly, when the ratio reached 1:5, some HSQC peaks started to reappear, and the reappearance was completed at 1:10. Further addition of TssDNA up to 1:50 showed no further changes. Interestingly, the HSQC spectrum of FUS in the presence of TssDNA at 1:10 is highly superimposable to that of the FUS NTD in the presence of ssDNA alone at 1:5. Therefore, the intensity of HSQC peaks of FUS without significant overlap was analyzed in the absence and in the presence of TssDNA at different ratios. Very interestingly, the PLD residues have their intensity slightly increased at a ratio of 1:0.1 and further increased at 1:0.5 (I of Fig. 2D). By contrast, the peak intensity of the RGG1, RRM, RGG2, ZnF, and RGG3 regions uniformly decreased. Most strikingly, at 1:10, almost all peaks of RRM, RGG2, ZnF, and RGG3 residues remained undetectable, but the peaks of PLD and some RGG1 residues reappeared. The intensity of most PLD residues in the presence of ssDNA at 1:10 is even slightly higher than that in the free state (II of Fig. 2D). To assess whether ATP and nucleic acids also share the same binding sites in the context of full-length FUS, ATP was added into an FUS sample to 3 mM, where the HSQC peak intensity was significantly reduced. Subsequently, TssDNA or T24 was gradually added. Upon adding TssDNA at a ratio of 1:10, corresponding to 0.2 mM of TssDNA, many HSQC peaks reappeared, and the reappearance became complete at 1:15 (0.3 mM). Further addition up to 1:50 showed no significant changes. Noticeably, the HSQC spectra of FUS in the presence of both ATP at 3mM and TssDNA at 0.3 mM are highly superimposable to that of the FUS NTD in the presence of TssDNA alone at 1:5. This observation implies that: 1) the residues of full-length FUS binding to ATP and TssDNA are the same or at least highly overlapped; and 2) the binding affinity of TssDNA to these residues is much higher than that of ATP, and consequently, TssDNA at 0.3 mM could displace ATP at 3 mM from binding with FUS.

Hence, through their binding to similar residues, ATP and nucleic acids modulate LLPS of the full-length FUS molecule (I of Fig. 2E) in the same manner: enhancement at low concentrations and dissolution at high concentrations (Fig. 2E). ATP achieves this modulation through bivalent binding, utilizing its adenine aromatic rings to establish π-π and π-cation interactions with Arg residues and π-cation interaction with Lys residues. Additionally, the phosphate oxyanion of the triphosphate group establishes electrostatic interactions with Arg/Lys residues (II and III of Fig. 2E). Similarly, nucleic acids achieve the same biphasic modulation by utilizing their base aromatic rings and phosphate oxyanion to establish the multivalent binding to Arg/Lys residues (II' and III' of Fig. 2E).

**Figure 3.**
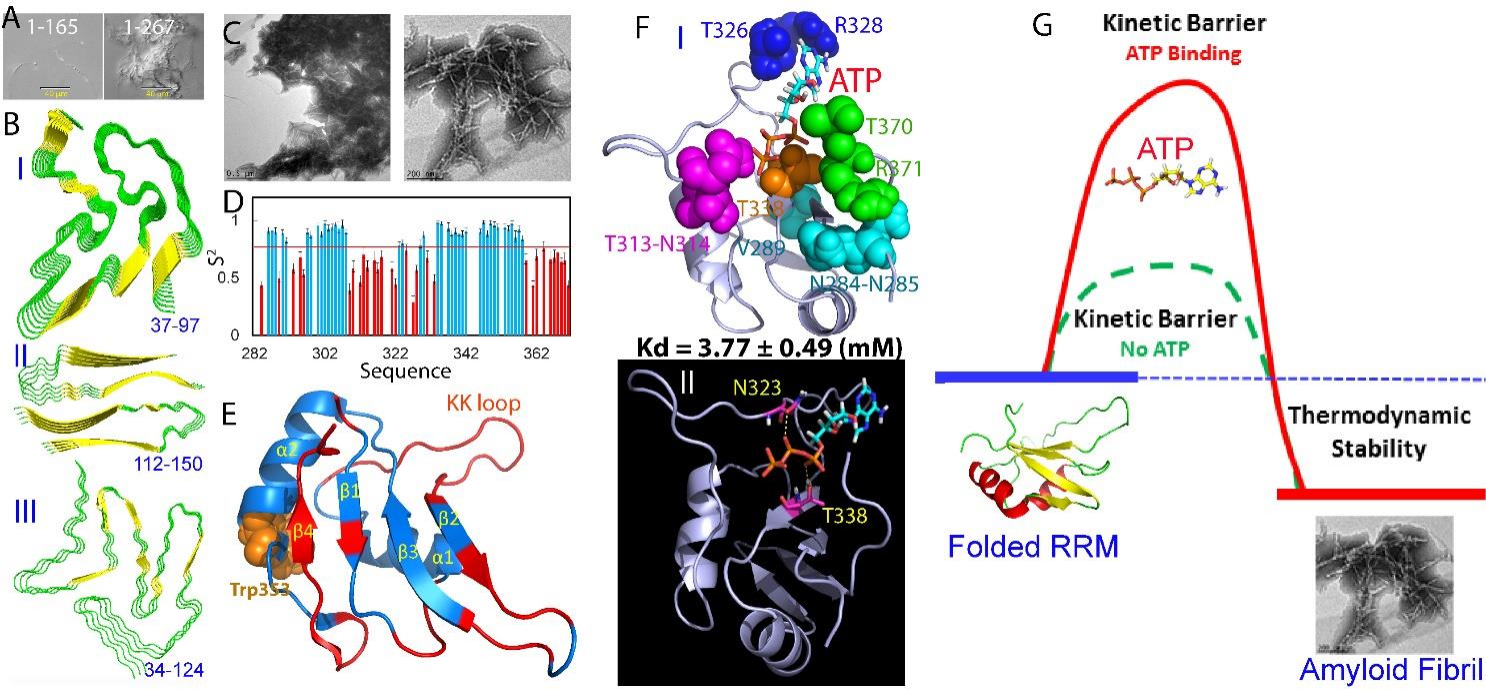
Amyloidosis of intrinsically-disordered regions and RRM of FUS. (A) EM images of the amyloid fibrils formed by FUS PLD (1-165) and NTD (1-267). (B) (I) Solid-state NMR structure of residues 37-97, viewed down the fibril growth axis, illustrating the overall fold; (II) Cryo-EM structures of amyloid fibrils formed over residues 112-150. (III) Cryo-EM structures of amyloid fibrils formed over residues 34-124. (C) EM images of the amyloid fibrils formed by FUS RRM. (D) Generalized squared order parameter (S^2^) of the FUS RRM domain. Light blue bars indicate residues with S^2^ > 0.77 while red bars for residues with S^2^ < 0.77 (average value as displayed as red line). (E) NMR structure of the FUS RRM domain (pdb ID of 2LCW) with residues having S^2^ < 0.77 colored in red. (F) FUS RRM domain in complex with ATP. (G) A proposed diagram to illustrate that the specific ATP binding kinetically inhibit the self-assembly of FUS RRM into amyloid fibrils without detectable alteration of its thermodynamic stability.

### 2.3. Amyloidosis of FUS domains

A systematic screening revealed that FUS NTD and RRM spontaneously formed amyloid fibrils in vitro at room temperature, whereas CTD showed no such ability even with prolonged incubation [78,79]. Interestingly, FUS PLD (1-165) had a requirement for high concentrations (>50 μM) in order to undergo phase separation and form liquid droplets, which could subsequently transform into a hydrogel state containing amyloid-like fibrils following a long incubation [86,87]. Conversely, FUS NTD (1-267) exhibited the ability to undergo phase separation at low concentrations (~1 μM), with a rapid transition into fibrils (Fig. 3A). Solid-state NMR analysis revealed that within the self-assembling fibrils formed by FUS (1-214), only a segment of 57 residues (39-95) constituted the fibril core, while other regions remained dynamically disordered [91]. Within the fibrils, residues 44-46, 52-54, 62-64, 67-70, 85-90, and 93-95 formed β-strands that adopted a cross-β motif, characterized by the alignment of backbone carbonyl groups with the fibril growth axis (I of Fig. 3B). In contrast to the tightly packed steric zippers found in amyloid fibrils (103-105), the interaction between these kinked sheets was weak, primarily involving polar atoms and aromatic side chains. These unique properties were further revealed in the crystal structures of several short segments of FUS NTD (Fig. 2A) [91]. Moreover, a cryo-EM structure of FUS NTD residues 112-150 revealed the adoption of U-shaped conformations, forming two subunits with in-register, parallel cross-β structures (II of Fig. 3B) [106]. The fibril core was found to be stabilized through a multitude of hydrogen bonds involving sidechains of Gln, Asn, Ser, and Tyr residues, both along and transverse to the direction of fibril growth. These interactions included diverse sidechain-to-backbone, sidechain-to-sidechain, and sidechain-to-water interactions. NMR measurements further demonstrated that portions of the disordered residues 151-214 exhibited high dynamics within the fibrils [91]. Very recently, a cryo-EM structure revealed a novel serpentine fold assumed by FUS NTD (34-124). This fold comprised three motifs that came together through a Tyr triad, resulting in the formation of an enlarged and stable fibril core (III of Fig. 3B). The stabilization of this fibril core was achieved through hydrophilic interactions and hydrogen bonds [107], which distinguished it from the commonly observed hydrophobic interaction-stabilized fibrils in previous studies [103-105].

Intriguingly, in vivo studies have shown that FUS cytotoxicity requires the presence of RRM [108], suggesting a crucial role for RRM domain fibrillation in the observed "gain-of-toxicity" in FUS. Spontaneous assembly of FUS RRM into amyloid fibrils has been observed under native conditions at room temperature (Fig. 3C) [78]. These findings imply that the amyloid fibrils formed by FUS PLD, lacking significant hydrophobic residues and relying primarily on interactions among polar residues, are relatively less toxic compared to the fibrils formed by FUS RRM, which contains numerous large hydrophobic residues.

To understand the biophysical basis for amyloidosis of FUS RRM, ^15^N NMR backbone dynamics of FUS RRM were obtained which exhibit substantial conformational dynamics on ps-ns time scale. Briefly, the squared generalized order parameters, S^2^, displayed in Fig. 3D, depict the ps-ns conformational dynamics. S2 values vary between 0, indicating significant internal motion, and 1, signifying complete motion restriction within a molecular reference frame. The average S^2^ value for FUS RRM is 0.77, significantly lower than that of EphA4 (0.86), a typical well-folded protein [109]. Specifically, the N- and C-terminal residues, along with the majority of residues over loops/turns, notably the unique KK-loop, exhibit S^2^ values below the average. Remarkably, even several residues situated within the β1, β2, and β3 strands display S^2^ values lower than the average. All C-terminal residues starting from Phe359, including the entire β4 strand, also demonstrate S^2^ values smaller than the average (Fig. 3E).

To gain insights into the biophysical basis of FUS RRM amyloidosis, ^15^N NMR measurements were utilized to investigate the backbone dynamics of FUS RRM. The obtained results revealed significant conformational dynamics of FUS RRM on the ps-ns timescale. The squared generalized order parameters, S2, depicted in Figure 3D, were employed to illustrate the ps-ns conformational dynamics. S2 values range from 0, indicating unrestricted internal motion, to 1, indicating complete motion restriction within a molecular reference frame. The average S2 value for FUS RRM was found to be 0.77, which is notably lower than that of EphA4 (0.86), a representative well-folded protein [109]. Notably, the N- and C-terminal residues, as well as the majority of residues located in loops/turns, particularly the unique KK-loop, exhibited S2 values below the average. Interestingly, even several residues within the β1, β2, and β3 strands displayed S2 values lower than the average. Additionally, all C-terminal residues starting from Phe359, including the entire β4 strand, exhibited S2 values smaller than the average (Fig. 3E).

Considering the high concentrations of ATP in living cells, a question arises regarding whether ATP can interact specifically with FUS RRM and influence its amyloid fibril formation. In a recent study [110], the interactions between FUS RRM and ATP, AMP, and triphosphate (PPP) were investigated using NMR. Interestingly, ATP was found to bind specifically to a pocket located on the nucleic-acid-binding surface of FUS FFM, with a Kd value of 3.77 ± 0.49 mM. This pocket comprises 10 residues situated across the N- and C-termini and loops, except for Val289 within the first β-strand and Thr338 within the third β-strand (I of Fig. 3F). Both the adenine aromatic ring and the triphosphate chain of ATP were observed to contribute to the interaction with FUS RRM residues. The aromatic ring of ATP is positioned within a relatively hydrophobic pocket, while the triphosphate chain is embedded in a pocket of FUS RRM with a negatively charged surface. Specifically, the aromatic ring of ATP directly contacts the side chain of Arg328, likely through π-π and π-cation interactions [111], while the oxygen atoms of the triphosphate chain form hydrogen bonds with the side chain protons of Asn323 and Thr338, respectively (II of Fig. 3F). Surprisingly, despite not significantly affecting the thermodynamic stability of FUS RRM, the presence of ATP at a concentration of 3 mM was found to inhibit the formation of amyloid fibrils. This inhibition was demonstrated by multiple biophysical probes, including ThT-binding induced fluorescence and electron microscopy [110].

Therefore, it appears that ATP binding inhibits amyloidosis by increasing the kinetic barrier (Fig. 3G). In brief, FUS RRM consists of a central four-stranded β-sheet flanked by two α-helices on one side and the N- and C-termini along with loops on the other side. Previous studies have shown that FUS RRM exhibits relatively high backbone dynamics even on the ps-ns timescale (Fig. 3D and 3E). Consequently, the self-assembly of FUS RRM into amyloid fibrils is likely initiated by the dynamic opening of the structure, allowing for intermolecular oligomerization driven by the central β-sheets. Thus, the fibrillation process of the free FUS RRM domain has a relatively low kinetic barrier. Interestingly, the ATP-binding pocket of the RRM domain is predominantly formed by the N- and C-termini and loops. Therefore, the binding of ATP into this pocket is expected to partially restrict the structure opening, leading to an increase in the kinetic barrier for fibrillation [110]. These studies offer a potential explanation for the requirement of RRM in FUS cytotoxicity [108]. The fibrils formed by FUS PLD, being primarily stabilized by weaker polar interactions, are reversible in nature. In contrast, RRM fibrillization is driven by hydrophobic interactions, which are irreversible and associated with higher toxicity. This distinction suggests that the irreversible and highly toxic nature of RRM fibrils contributes to the observed cytotoxic effects of FUS.

**Figure 4.**
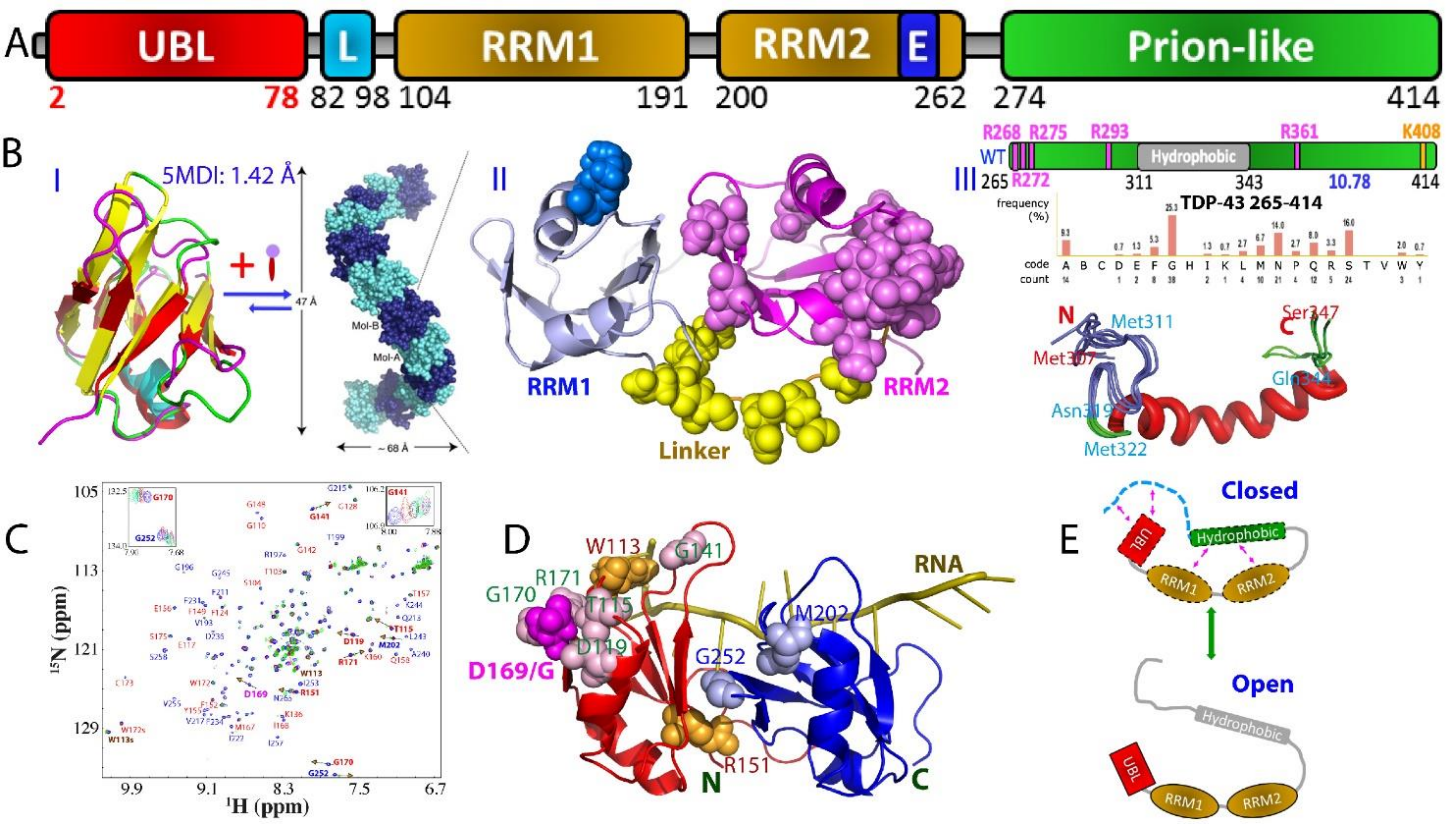
Domain organization, sequence and structures of TDP-43. (A) Domain organization of 419-residue human TDP-43 protein. (B) (I) Three-dimensional structures of NTD which can oligomerize into the helical filament with single molecules arranged in head-to-tail fashion particularly enhanced by binding ATP. (II) linked RRM1 and RRM2 showing the correlated residues between two RRM domains identified by NMR. (III) Amino acid compositions of intrinsically-disordered PLD (265-414) and NMR structure of the hydrophobic fragment 307-347 in the membrane-mimicking environment. (C) Superimposition of HSQC spectra of the ^15^N-labeled TDP-43 RRM12 in the presence of the unlabelled PLD at molar ratio (PLD/RRMs) of 0 (blue), 0.5 (red) and 2.0 (green). Assignments of some significantly shifted peaks are labeled in red if within RRM1 or blue within RRM2. The shift tracings are indicated by arrows for 9 significantly shifted peaks. Inlets: the shift tracings of Gly141, Gly170 and Gly252 in the presence of the unlabelled TDP-43 PLD at 1:0 (blue), 1:0.5 (red), 1:1 (black), 1:1.5 (cyan), 1:2.0 (green) and 1:2.5 (purple). (D) NMR structure of TDP-43 RRM12 in complex with RNA (PDB ID: 4BS2) in which spheres are used to represent 9 residues with significantly shifted HSQC peaks as well as Trp113. D169G mutant is the only ALS-causing mutant identified so far on RRMs. (E) Diagram for illustrating the inter-domain interaction of TDP-43 and the exchange between the closed and open states.

## 3. TAR-DNA-binding protein-43 (TDP-43)

### 3.1. Domain organization, amino acid composition and structures of TDP-43.

TAR-DNA-binding protein-43 (TDP-43) was initially identified to bind TAR DNA in HIV to inhibit its transcription [112]. TDP-43 is a multifunctional protein from the hnRNP family, which is involved in various cellular processes. It primarily localizes in the nucleus but can shuttle between the nucleus and cytoplasm. Within the nucleus, TDP-43 binds to DNA and RNA, particularly with a high affinity for single-stranded DNA and RNA, encompassing over 6000 RNA species. It plays a crucial role in regulating RNA metabolism, including transcription, splicing, and mRNA stability [113]. Additionally, TDP-43 can undergo liquid-liquid phase separation, contributing to the formation of stress granules (SGs) and other membraneless organelles, which are vital for cellular stress response and RNA metabolism. Misfolding/inclusion of TDP-43 in the cytoplasm is a hallmark characteristic of neurodegenerative diseases such as ALS and FTD, leading to loss of normal nuclear function and acquisition of toxic properties that contribute to neuronal dysfunction and cell death [114-117]. The exact mechanisms underlying TDP-43 pathology involve impaired RNA processing and disruption of protein homeostasis. TDP43 has emerged as one of the most extensively studied proteins in neurodegenerative diseases and is also implicated in cancer, making it a crucial target for drug design in both fields [117,118].

The 414-residue TDP-43 is composed of distinct domains: the N-terminal domain (NTD) over residues 1-80, two RNA recognition motif (RRM) domains over residues 104-262, and a C-terminal PLD domain rich in Gln/Asn/Ser/Gly over residues 265-414 (Fig. 4A). Initially, due to its propensity for aggregation and lack of sequence homology with other proteins, TDP-43 NTD was widely believed to lack a structured domain. However, through the discovery that insoluble proteins can be solubilized in unsalted water [119] and the utilization of the CS-Rosetta method with sparse NMR data [120], the structure of TDP-43 NTD spanning residues 1-80 was first determined to adopt a fold similar to ubiquitin (I of Fig. 4B), which possesses the ability to bind RNA and single-stranded DNA [121]. So far, four more structures have been reported, including three NMR structures [122-124] and one crystal structure [125]. The first NMR structure exhibits the highest similarity to the crystal structure (PDB ID 5MDI), with a backbone RMSD value of only 1.42 Å (I of Fig. 4B). Intriguingly, further investigations have revealed that the structures of TDP-43 NTD are more akin to several proteins in the ubiquitin superfamily, such as aXin-1 and Dishevelled (DIX) domains, rather than ubiquitin itself [122-125]. Strikingly, TDP-43 NTD forms head-to-tail oligomers arranged as a tubular super-helical filament (I of Fig. 4B), representing its physiological and functional state in vivo [125]. This NTD-driven oligomerization of TDP-43 was proposed to serve to spatially separate the C-terminal PLD, which prone to aggregation and amyloid fibrillation, thereby reducing the intermolecular interactions between PLD monomers and counteracting the formation of pathological aggregates [125,126].

TDP-43 is also composed of two tandemly-tethered RNA recognition motif (RRM) domains (II of Fig. 4B), which play a crucial role in binding various nucleic acids, including single- or double-stranded DNA/RNA, and contribute to a range of functions such as transcriptional repression, pre-mRNA splicing, translational regulation [127-129], and even muscle regeneration [130]. The crystal structures of the individual RRM1 and RRM2 domains have been determined separately [127, 128], while the tandem RRM1 and RRM2 domains in complex with RNA were elucidated using NMR techniques [129]. Structurally, both RRM domains of TDP-43 adopt the characteristic fold observed in all RRM domains, consisting of a four-stranded β-sheet and two perpendicular α-helices. However, unlike the RRM domain of FUS, the RRM domains of TDP-43 do not possess the "KK-loop," distinguishing them structurally from other members of the RRM family [127,129].

Interestingly, the tethered RRM1-RRM2 in TDP-43 exhibits a notable propensity for aggregation, while the isolated domains have remarkable stability and solubility [131, 132]. Moreover, the tethered RRM1-RRM2 experiences significant destabilization, as indicated by a reduced melting temperature (Tm) of only 49 °C, whereas the isolated RRM1 and RRM2 domains have higher Tm values of 57 °C and 59 °C, respectively [131-133]. Surprisingly, under the same experimental conditions, the tethered RRM1-RRM2 can form amyloid fibrils within a few days, whereas the isolated domains fail to fibrillate even after a month [133]. Recent studies employing NMR and molecular dynamics (MD) simulations have revealed that the inter-domain correlation motions exist between two TDP-43 RRM domains which are strongly coupled (II of Fig. 4B), providing a rational explanation for these observations [131].

TDP-43 PLD plays a crucial role in both driving phase separation and associating with ALS pathogenesis. This connection is particularly evident due to the fact that TDP-43 PLD harbors nearly all known ALS-caused mutations and has been found to be responsible for the prion-like propagation of ALS. Intriguingly, unlike prototypic PLD domains such as FUS PLD (1-165) which is rich in aromatic residues (I of Fig. 1B), the TDP43 PLD is distinguished by the inclusion of 10 methionine residues and a 25-residue region which are evolutionarily ultra-conserved in vertebrates (III of Fig. 4B) [134-136]. However, due to the inherent tendency of the full-length PLD to aggregate, NMR investigations have primarily focused on its dissected fragments [137,138]. Recent NMR studies have revealed that the full-length TDP-43 PLD is intrinsically disordered [22,139], with only a nascent helical conformation observed in a hydrophobic region from Ser403 to Met414 at acidic pH which, however, could transform into β-like conformation at neutral pH [22]. Interestingly, a membrane-interacting subdomain spanning residues Met311 to Gln343 has been identified, which is essential for TDP-43 neurotoxicity. This subdomain undergoes a conformational transformation into a well-folded Ω-loop-helix structure (III of Fig. 4B) when embedded in membrane environments [22].

A crucial question arises as whether the inter-domain interactions exist in TDP-43 [140]. Understanding these interactions is vital in utilizing the structural information acquired from isolated domains to comprehend the properties of full-length TDP-43. The presence of inter-domain interactions, if existent, could significantly impact the aggregation mechanism and biochemical characteristics of TDP-43, including its interaction with nucleic acids. Furthermore, this knowledge is highly critical for gaining insights into the physiology and proteinopathy of TDP-43 within cells, as the modulation of inter-domain interactions might be tightly regulated by cellular processes. Notably, serine phosphorylation has been implicated in TDP-43 pathogenesis, yet the underlying molecular mechanism remains elusive [140-142].

In contrast to the full-length FUS, which exhibited detectable HSQC peaks for most residues of all domains, an unexpected observation was made with full-length TDP-43. Not only were the HSQC peaks of the N-domain and two RRMs undetectable, but the majority of HSQC peaks from the prion-like domain also vanished [140]. Consequently, the interactions between domains were further characterized using NMR with differentially dissected domains. For instance, ^15^N-labeled RRM1-RRM2, connected together, was titrated with unlabeled PLD (Fig. 4C). Nine residues in RRM1 and RRM2 were identified to interact with PLD, including Thr115, Asp119, Gly141, Arg151, Asp169, Gly170, and Arg171 in RRM1, as well as Met202 and Gly252 in RRM2 (Fig. 4D), with estimated Kd values of approximately 5 μM. It is possible that the binding affinity might be higher and/or the interacting interface may be larger when the RRMs and prion-like domain are covalently linked, as in the case of TDP-43 (102-414), where all HSQC peaks of the RRMs disappeared. Interestingly, in the full-length TDP-43, only HSQC peaks corresponding to a limited region preceding the hydrophobic fragment were observable [140]. This region includes residues Gly277, Gly278, Gly281, G282, Gly284, Gly287, Gly288, Gly290, Gly294, Gly295, and Gly298. These observations suggest that the prion-like domain interacts with both the N-domain and RRMs. Furthermore, residues within the prion-like domain, in addition to the N-terminal region spanning Gly277-Gly298, are involved in interactions with the RRMs and N-Domain. These interactions induce microsecond-millisecond dynamics, ultimately leading to the loss of HSQC peaks for a significant portion of TDP-43 residues, similar to what has been extensively observed on the interactions involved in intrinsically-disordered proteins [143].

Therefore, inter-domain interactions do exist in TDP-43 but have relatively low affinity (in the micromolar range), resulting in conformational exchange between "closed" and "open" states on a microsecond to millisecond timescale (Fig. 4E). These conformational dynamics appear to be essential for TDP-43 to fulfill its functions. On one hand, these interactions can help prevent excessive exposure of the N-domain and prion-like domain, thereby minimizing their aggregation and toxicity, as recently discovered. On the other hand, if needed, the conformational equilibrium can be shifted towards the open state, which is favorable for binding its ligands such as partner proteins, phase separation, and reversible self-assembly into functional oligomers. Due to the presence of inter-domain interactions, the full-length TDP-43 is expected to exhibit distinct features compared to individual domains. Indeed, under pathological conditions, inter-domain interactions can be disrupted, for example, through ALS-causing cleavage of RRM2. This disruption dramatically facilitates the formation of inclusions and amyloid fibrils characteristic of ALS pathogenesis [144].

### 3.2. LLPS of TDP-43.

LLPS of the full-length TDP-43 has been shown to be driven by multivalent interactions involving different domains, including the folded NTD and intrinsically disordered PLD. NTD oligomerization is proposed to play a critical role in the assembly of TDP-43 into head-to-tail linear chains responsible for LLPS [125,126]. However, the existence of dynamic inter-domain interactions makes it extremely challenging to characterize the full-length TDP-43 LLPS using high-resolution NMR methods [140]. Consequently, NMR studies have mostly focused on dissected TDP-43 domains. In the absence of molecular crowding agents such as polyethyleneglycol (PEG), only the isolated TDP-43 PLD demonstrates the ability to undergo LLPS [22,134-136,139,145-150]. On the other hand, the isolated NTD (1-102), linked RRM12 (102-269), and NTD-RRM12 (1-269) lack the capability to phase separate individually. However, the addition of 10% w/v PEG-3350 induces LLPS of TDP-43 RBDs (1-273) at 50 μM, which was proposed to primarily rely on the oligomerization of the N-terminal domain (NTD) [151].

**Figure 5.**
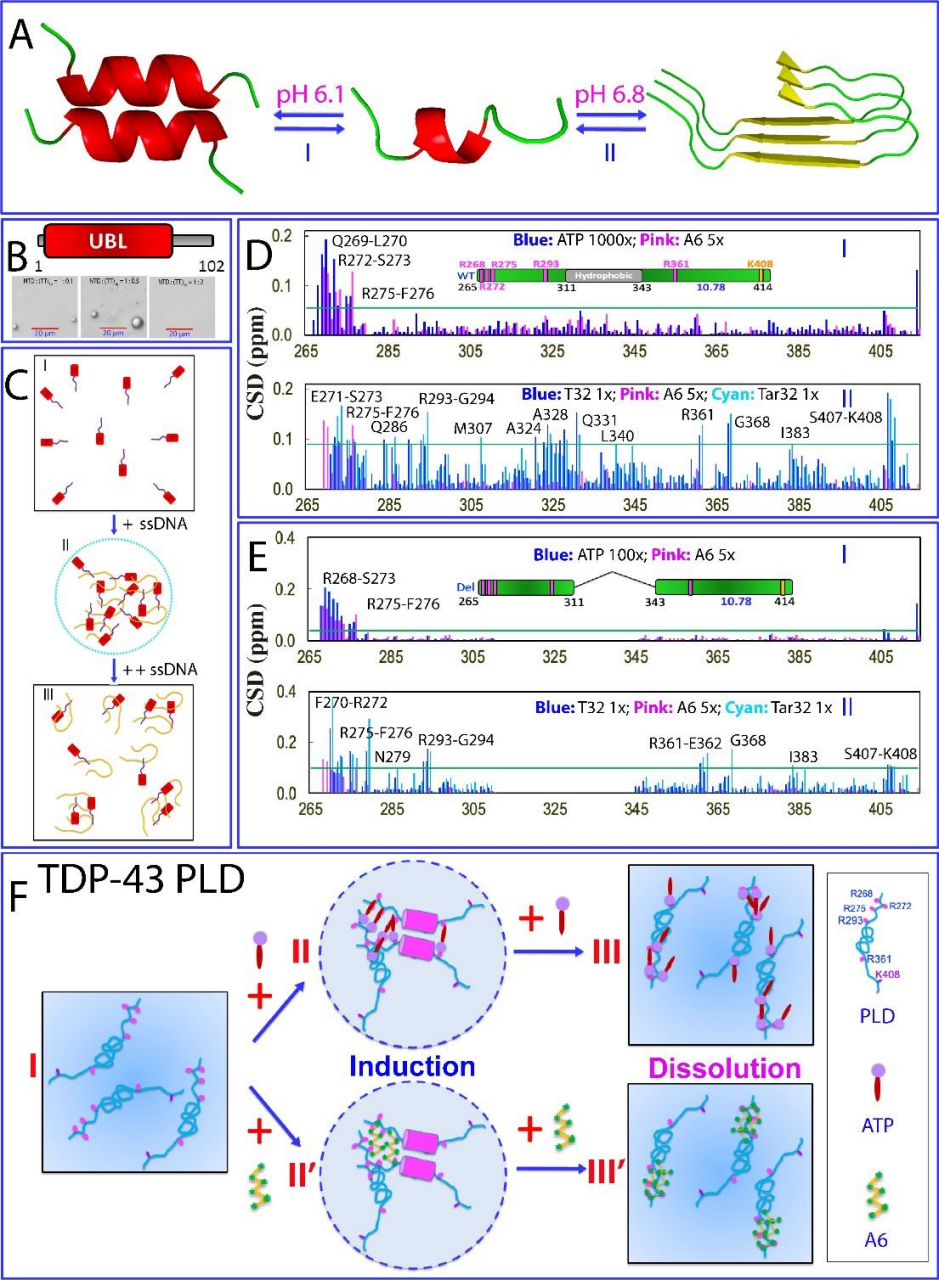
LLPS and its modulation by ATP and nucleic acids of TDP-43 Domains. (A) Two proposed mechanisms for LLPS of TDP-43 PLD. (B) ssDNA biphasically modulates LLPS of TDP-43 NTD (1-102) consisting of well-folded domain over 1-80 and intrinsically-disordered region 81-102. (C) A speculative model to rationalize the specific binding of ssDNA to induce and subsequently dissolve LLPS of NTD. (D) Chemical shift differences (CSDs) of HSQC peaks of WT-PLD upon addition of ATP and A6 (I); A6, Tar32 and T32 (II). (E) CSDs of HSQC peaks of Δ(311-343)-PLD upon addition of ATP and A6 (I); A6, Tar32 and T32 (II). (f) A speculative model for ATP and ssDNA to modulate LLPS of TDP-43 PLD.

With regard to TDP-43 PLD, extensive studies revealed that the evolutionarily conserved hydrophobic region over residues 311-343 with the nascent helical conformation plays a key role in driving LLPS [134-136,139,145-150]. Indeed, TDP-43 PLD with residues 311-343 deleted loses the ability to phase separate under various solution conditions in vitro [149,150]. Interestingly, one residue-specific NMR study at pH 6.1 [139] suggested that LLPS of TDP-43 PLD primarily occurs through intermolecular self-interaction of residues 321-340 to promote the helical propensity (I of Fig. 5A). Additionally, the spacing of hydrophobic ‘sticker’ residues interspersed by flexible linkers, electrostatic interaction and aromatic residues adjacent to Gly or Ser have also been proposed to be important [146-148]. Interestingly, other studies suggested that at pH 6.8 the nascent helical conformation of residues 321-340 undergoes transformation into the labile cross-β oligomer (II of Fig. 5A), which facilitate biologically-relevant LLPS and amyloidosis [134-136,152]. Evidently, oxidation of methionine residues, which enhances the helical conformation, hinders the cross-β assembly as well as LLPS [134,135]. In this context, the variation in pH values among these studies appears to critically account for the conformational differences observed on this evolutionarily-conserved region. Indeed, acidic pH values below pH 6.5 were found to largely inhibit the α-to-β transformation as well as LLPS and further amyloidosis of TDP-43 PLD [22, 134-136, 152].The cellular environments where TDP-43 exists is rich in ATP and nucleic acids [153-156]. A recent *in vivo* study revealed distinct pathways by which LLPS of TDP-43 is regulated through its oligomerization and interactions with RNA and ATP [126]. TDP-43 appears to undergo alternation between NTD- and PLD-mediated LLPS, determined by the presence or absence of RNA and ATP. NTD-mediated oligomerization and RNA binding maintain TDP-43's localization, LLPS, and physiological function. Disruption results in nuclear and cytoplasmic aggregation observed in ALS and FTLD. As the direct binding of ATP to TDP-43 NTD promotes its functional oligomerization [156], reduced cellular ATP levels with age [157] might induce monomerization, causing TDP-43 to exit the nucleus into the cytoplasm, where aberrant LLPS and aggregation may occur. Additionally, even in the absence of specific RRM-RNA interactions, high RNA concentrations within the nucleus inhibit or dissolve LLPS of RNA-binding proteins like TDP-43 [96], thus acting as a protective measure against abnormal LLPS and aggregation. Consequently, an important question arises: do ATP and nucleic acids provide additional driving forces for the LLPS of TDP-43 domains?

A study using 10 ssDNA sequences, including functional ligands Tar32 and non-specific sequences of various lengths, revealed that ssDNA can biphasically modulate LLPS of the NTD (1-102) of TDP-43 consisting of well-folded residues 1-80 and an intrinsically disordered region 81-102. At low concentrations of ssDNA, NTD was induced to phase separate, while at high concentrations, LLPS was dissolved (Fig. 5B). In contrast, no LLPS was induced when ssDNA was added to NTD (1-80) [153]. Further studies [156] found that ATP could bind NTD (1-102) over both well-folded residues 1-80 and intrinsically disordered region 81-102 but induced no LLPS. Based on these findings, a model was proposed for the ssDNA-induced LLPS of NTD (1-102) (Fig. 5C). Briefly, the folded TDP-43 NTD has been shown to weakly bind both RNA and ssDNA using a large positively-charged surface [121]. Additionally, two Arg/Lys-rich regions within the disordered region 81-102, could also weakly interact with ssDNA [122]. Thus, the TDP-43 NTD (1-102) contains multiple sites for weak interactions with nucleic acids. As such, in the absence of ssDNA, the TDP-43 NTD (1-102) exists as a homogeneous solution (I of Fig. 5C). However, upon adding ssDNA, dynamic heterotypic assemblies of ssDNA-NTD (1-102) can form through weak and multivalent interactions, manifesting as liquid droplets (II of Fig. 5C). The formation of these liquid droplets depends on the number of binding sites on the ssDNA, making the capacity of ssDNA to induce LLPS length-dependent. Furthermore, if the molar ratio between ssDNA and NTD (1-102) is too high, each ssDNA molecule can only bind one or a small number of NTD (1-102) molecules, as well as the ssDNA-NTD complex become highly negatively-charged and thus repulsive (III of Fig. 5C). As a result, the large and dynamic heterotypic assemblies is restricted, leading to the reduction or dissolution of the liquid droplets. Moreover, Poly(ADP-Ribose) (PAR), which structurally resembles nucleic acids, has also been shown to induce LLPS of TDP-43 NTD (1-102), likely through similar binding to the folded NTD (1-80) and two PAR-binding motifs (PBMs) containing Arg/Lys residues within the disordered region [154].

As shown in Fig. 2C, for FUS CTD which is intrinsically-disordered and rich in RG-/RGG-motifs, its LLPS was shown to be biphasically modulated through the binding of ATP and nucleic acids to CTD Arg/Lys residues. However, the high abundance of Arg/Lys residues within FUS CTD (25 Arg and 4 Lys) makes it challenging to determine, through NMR and site-directed mutagenesis, the high-resolution mechanism for ATP and RNA/DNA specifically to bind these Arg/Lys residues for modulating LLPS. In this context, TDP-43 PLD (265-414) only has a small number of Arg and Lys residues, specifically Arg268, Arg272, Arg275, Arg293, Arg361, and Lys408 (I of Fig. 5D), providing an excellent model to explore whether LLPS of TDP-43 PLD can be modulated by ATP and nucleic acids. If such modulation occurs, does it happen through non-specific electrostatic/salt effects or specific binding of ATP and nucleic acids to Arg/Lys residues?

Recently, two studies have systematically investigated the effects of ATP and ssDNA on LLPS of TDP-43 PLD as well as the residue-specific interactions by DIC and NMR [149,150]. Briefly, these investigations focused on effects of ATP, ssDNA including Tar32, A32, and A6 on LLPS as well as their molecular interactions with WT (Fig. 5D) and mutant PLDs including PLD Δ(311-343) with residues 311-343 deleted but pI unaltered (Fig. 5E), as well as All-K PLD with all five Arg residues replaced by Lys and pI of 9.6. Strikingly, ATP could biphasically modulate LLPS of TDP-43 PLD: induction at low ATP concentrations followed by dissolution at high concentrations [149]. Very unexpectedly, ATP only induces the shift of a small set of HSQC peaks upon addition even up to 1:1000. Briefly, except for the last residue Met414, the residues with significant shifts are clustered around N-terminal three Arg residues, which include Arg268-Gln269-L270-Glu271-Arg272-Ser273 and Arg275-Phe276 (I of Fig. 5D). Furthermore, for a PLD mutant with Arg268, Arg272, and Arg275 mutated to Ala, ATP was still able to induce LLPS but with much less droplet number. NMR characterization revealed that the residues clustered over three N-terminal Arg residues no longer showed large shifts, indicating that the observed shift resulted from specific binding of ATP to three Arg [149]. For PLD Δ(311-343) without the ability to phase separate under various solution conditions, the addition of ATP even upon to 1:1500 also failed to induce its LLPS or aggregation [149]. Nevertheless, ATP still induced significant shifts of N-terminal residues (I of Fig. 5E) whose pattern is very similar to that of the WT PLD (I of Fig. 5D). For All-K PLD, the addition of ATP could only slightly induce LLPS while further addition of ATP only led to a slow dissolution of the droplets. On the other hand, upon adding ATP, All-K PLD has an overall shift pattern of HSQC peaks very similar to that of WT. Nevertheless, for WT PLD, the shift of HSQC peaks was saturated at 1:100 (PLD:ATP), while for All-K PLD, the shifts remained unsaturated even at 1:500 [149]. The results unambiguously unveiled that ATP specifically binds Arg residues regardless of the sequence contexts of different TDP-43 PLD mutants, with the binding affinity much higher than that of Lys, consistent with previous NMR studies [158]. Furthermore, unlike FUS CTD abundant in Arg/Lys residues whose LLPS was solely modulated by ATP, for TDP-43 PLD, whether ATP induces LLPS further depends on the presence of the unique region 311-343, although ATP bind the same set of residues of WT and Δ(311-343) PLD.

Therefore, due to its weak binding affinity, the exact mechanisms for the ATP binding to modulate LLPS appear to be highly context-dependent. For 156-residue FUS CTD with 25 Arg and 4 Lys residues, the bivalent binding of ATP to Arg and Lys is sufficient to directly induce LLPS by forming large and dynamic complexes at low ATP ratios followed by dissolution at high ATP ratios due to the exceedingly binding (Fig. 2C). By contrast, for 150-residue TDP-43 PLD with only 5 Arg and 1 Lys residues, the ATP binding is insufficient to solely drive LLPS, and thus needs to coordinate other driving forces, particularly the oligomerization of the unique hydrophobic region to induce LLPS (Fig. 5F).

In another study [150], three ssDNA have been shown to biphasically modulate LLPS of TDP-43 WT PLD. For A6, LLPS reached the highest at a molar ratio of 1:3, whereas the dissolution was completed at 1:5. On the other hand, upon stepwise addition of Tar32, LLPS reached the highest at 1:0.25. Further addition of Tar32 led to dissolution of LLPS at 1:1. A32 also induced and then dissolved LLPS with a pattern very similar to that of Tar32. NMR characterization revealed that the addition of A6 triggered the shift of only a small set of HSQC peaks, whose pattern is very similar to that induced by ATP (I of Fig. 5D). By contrast, the addition of Tar32 induced the shift of a large set of HSQC peaks from the residues over the whole PLD sequence, while the addition of A32 led to a shift pattern very similar to that of Tar32 (II of Fig. 5D).

Noticeably, for PLD Δ(311-343), A6 could trigger the shift of a small set of HSQC peaks very similar to that induced by ATP (I of Fig. 5E), but was unable to induce LLPS with the ratio even up to 1:5. By contrast, Tar32 induced LLPS and reached the highest at 1:0.25, while at 1:1 LLPS was completely dissolved. Interestingly, A32 modulated LLPS with a pattern very similar to that by Tar32. Further NMR studies indicated that the residues significantly perturbed by Tar32 and A32 are very similar (II of Fig. 5E). The results indicate that the existence of the hydrophobic region 311-343 appears to have no detectable impact on the binding affinity of A6, A32, and Tar32 to Arg/Lys residues. Nevertheless, it has a key contribution to the strength of the driving force for LLPS. For ATP and A6 with the low binding affinity, although they still bind the similar set of residues of Del-PLD at similar affinity, they failed to induce LLPS because of the absence of the intrinsic driving force from the oligomerization of residues 311-343. Nevertheless, for Tar32/A32 with a strong binding affinity, their multivalent binding is sufficient to drive and then dissolve LLPS of Del-PLD even without the intrinsic driving force.

The results together reveal that: 1) ATP, A6, Tar32, and A32 appear to biphasically modulate LLPS through commonly binding Arg/Lys residues because they are all composed of the same building unit: nucleotide. As such, they are all able to establish electrostatic interactions between the phosphate group of nucleotide and side chain cations of Arg/Lys as well as π-π/π-cation interactions between base aromatic rings and Arg/Lys side chains. 2) The results that Tar32 containing all four bases but A32 consisting of only adenine have highly similar modulating capacity suggest that four bases have a highly similar affinity in establishing π-π/π-cation interactions with side chains of Arg/Lys residues within IDRs. 3) Tar32 and A32 have the binding affinity much higher than those of ATP and A6, because ATP, A6, Tar32/A32 have different numbers of covalently linked nucleotides, and they are expected to have length-dependent affinities. It is well established that for a multivalent binder, its dissociation constant (Kd) value is the time of Kd values of the individual binding events if assuming these binding events are independent [102]. 4) The binding affinity of ATP and ssDNA to Arg is much higher than that to Lys because the base aromatic rings can establish π-π/π-cation interactions with Arg side chains, but only π-cation interaction with Lys side chains.

**Figure 6.**
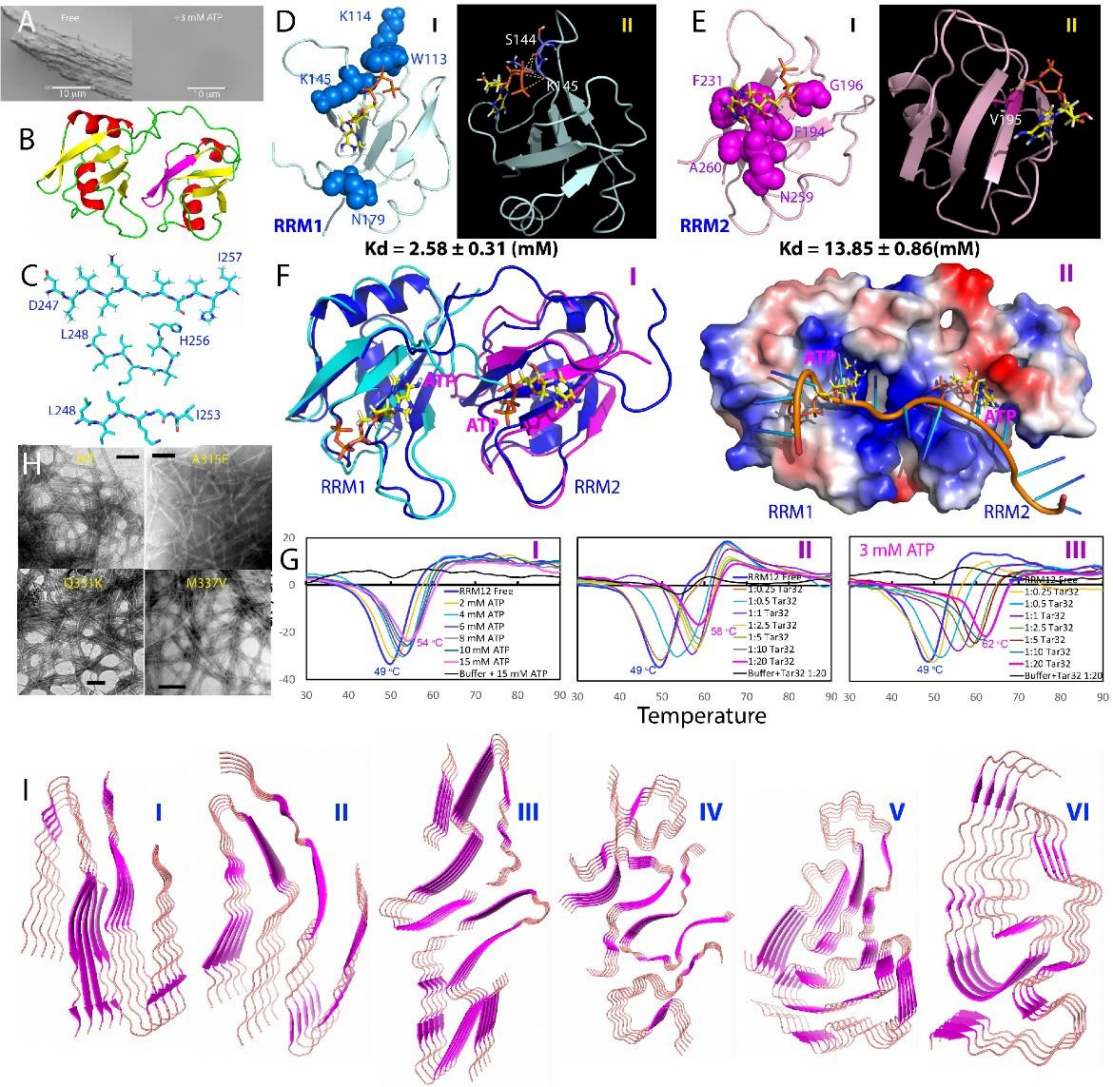
Amyloidosis of RRM1-RRM2 and PLD of TDP-43. (A) Amyloid formation of the linked RRM1-RRM2 domains without and with ATP at 3 mM. (B) Structure of RRM1-RRM2 with the RRM2 fragment 247-257 colored in purple. (C) Three different crystal structures of the amyloid fibrils formed by the RRM2 fragment 247-257. (D) Structures of RRM1 in complex with ATP. (E) Structures of RRM2 in complex with ATP. (F) Comparison of structures of RRM1-RRM2 in complex with ATP and with RNA in ribbon (I) and electrostatic surfaces (II). (G) DSF melting curves of thermal unfolding of the TDP-43 RRM1-RRM2 in the presence of ATP and Tar32 ssDNA at different concentrations, as well as with the pre-existence of 3 mM ATP and further addition of Tar32 ssDNA, by plotting the first derivative of the fluorescence emission as a function of temperature (dF/dT). Here, the Tm is represented as the lowest point of the curve. (H) Amyloid fibrils formed by WT PLD (265-414) and its three ALS-causing mutants A315E, Q331K and M337V. (i) Cryo-EM structures of various TDP-43 PLD fragments.

### 3.3. Amyloidosis of TDP-43 domains

Abnormal aggregation/amyloidosis of TDP-43 is a hallmark of ALS and FTD. In particular, TDP-43 was identified to undergo cleavages, resulting in different pathological fragments, out of which are two major groups: approximately 35 and 25 kDa fragments with elevated aggregation and cytotoxicity. The 25-kDa fragment, which is cleaved at Arg208, consists of the truncated RRM2 and CTD [125,140]. Extensive research has demonstrated that in addition to PLD, the two RRM domains of TDP-43 also play a crucial role in binding various nucleic acids, and contribute to disease-associated aggregation and fibrillation, especially when ALS-causing cleavage occurs [159-164]. Notably, recent discoveries have identified small molecules that target the RRM domains of TDP-43 and alleviate locomotor defects in a drosophila model of ALS [165].

Interestingly, the linked RRM1 and RRM2 became destabilized and was shown to form amyloid fibrils even under the native conditions at room temperature (Fig. 6A). The atomic structures of a fragment derived from residues 247-257 of RRM2 (Fig. 6B) have been determined. Strikingly, this fragment could form multiple amyloid polymorphs, with seven distinct interfaces involving five symmetry classes of steric zippers [166]. In particular, the fragment could adopt three distinct backbone conformations (Fig. 6C), showcasing the ability of an amyloid protein to generate diverse fibril structures at the molecular level [166].

As amyloid fibrillation of FUS RRM domain highly depends on its thermodynamic stability [78] and was modulated by binding to ATP [110], the binding of ATP to the linked TDP-43 RRM1 and RRM2 were characterized by NMR [131-133]. Both RRM1 and RRM2 could bind to ATP with the average dissociation constant (Kd) values of 2.58 ± 0.31 mM for RRM1 (Fig. 6D) and 13.85 ± 0.86 mM for RRM2 (Fig. 6E). This result indicates that although ATP is capable of specifically binding both RRM domains of TDP-43, it has significantly diverse affinities. As shown in Fig. 5F, the RRM1 and RRM2 structures in complex with ATP are very similar to those in complex with RNA. On the other hand, although ATP occupies the conserved pockets of RRM1 and RRM2 for binding nucleic acids, ATP and RNA binding sites are not completely overlapped (Fig. 6F). In the ATP-RRM1 complex, the triphosphate chain of ATP has close contacts with the side chains of K145 and K114 residues (Fig. 6D), while the aromatic ring of ATP inserts into a positively charged pocket constituted by K136, K137, K176 and K181. A close examination further reveals that the aromatic ring of ATP has close contacts with the side chains of both K176 and K181 by establishing π-cation interactions. Furthermore, three phosphate oxyanions of ATP form five hydrogen bonds with the backbone atoms of S144 and K145. By contrast, in the ATP-RRM2 complex, the aromatic ring of ATP has close contacts with the aromatic rings of F194 and F231 by establishing π-π interactions (Fig. 6E). Very different from that of the ATP-RRM1 complex, the ATP binding pocket on RRM2 appears to be even slightly negatively charged (II of Fig. 6F). Only one hydrogen bond is formed between the b phosphate oxyanions of ATP and the backbone atom of V195 (Fig. 6E). The very distinctive properties of the ATP-binding pockets of RRM1 and RRM2 thus explain their different binding affinities to ATP, and further reveal the general binding determinants for ATP-protein interactions at concentrations > mM: 1) π-cation interaction appears to be stronger than π-π interaction even in the well-folded RRM domains; 2) the number of the hydrogen bonds formed with the triphosphate chain appears to be also critical for the binding affinity because previously in the ATP-RRM complex of FUS, only one hydrogen bond is formed with the triphosphate chain. Consequently, its binding affinity (Kd of 3.77 mM) is slightly lower than the current ATP-RRM1 complex of TDP-43 (Kd of 2.58 mM), although the aromatic ring of ATP also established π-cation interactions with the FUS Arg residue with both π-π and π-cation interactions.

Very different from what was observed on FUS RRM domain [110], ATP can increase Tm of the linked TDP-43 RRM12 domains from 49 °C to 54 °C (I of Fig. 6G). Subsequently, Tar32, a functional ssDNA ligand of TDP-43 RRM domains significantly increase Tm of the linked RRM domains from 49 °C to 58 °C (II of Fig. 6g). Very interestingly, if with the preexistence of ATP at 3 mM which mimic ATP concentrations in neurons, Tar32 significantly increases Tm from 49 °C to 62 °C (III of Fig. 6G). Noticeably, ATP and Tar32 have additive effects in stabilizing the TDP-43 RRM domains, implying that Tar32 might not displace ATP from binding RRM domains, but instead Tar32 and ATP can synchronize in enhancing the stability, consistent with the observation that the binding pockets of RRM1 and RRM2 to ATP and RNA are only partly overlapped, and a close examination further reveals that ATP appears to insert into the binding cavity deeper than RNA due to its small size (Fig. 6F).

While during the incubation, the sample without ATP became cloudy and had HSQC peaks gradually disappeared, characteristic of a gradual increase in the fluorescence induced by ThT binding and formation of fibrils [133]. By contrast, the presence of ATP at 3 mM was sufficient to suppress the formation of fibrils even up to 15 days (Fig. 6A), thus implying that ATP at 3 mM is sufficient to inhibit amyloid fibrillation of TDP-43 RRM12 domains. Together with previous results with FUS [110], it appears that the binding of ATP to the RRM domains might represent a general safeguard mechanism to prevent “gain-of-toxicity” of RRM-containing proteins including, but not just limited to, TDP-43 and FUS. As such, the fact that neurons have relatively low ATP concentrations (~3 mM) may explain why TDP-43, FUS and other RRM-containing proteins are particularly prone to aggregation in the cytoplasm of neurons. Furthermore, it is well known that upon becoming aged, ATP concentrations in human cells including neurons become gradually reduced. The reduction of ATP concentrations might at least partly account for the long-standing observation that the risk of neurodegenerative diseases including ALS/FTD and AD significantly increases upon becoming aged. Strikingly, ATP can also serve as a promising starting molecule for further design of small molecules which are generally capable of inhibiting aggregation/fibrillation of proteins associated with an increasing spectrum of human diseases and ageing.

It is well-recognized that TDP-43 PLD is prone to forming amyloid fibrils and ALS-causing mutants enhance the fibrillation (Fig. 6H) [22]. Extensive efforts have attempted to determine the atomic-resolution structure of amyloid fibrils of various PLD fragments. In one study, bioinformatic approach was utilized to identify segments of the TDP-43 PLD responsible for TDP-43 aggregation and consequently 15 fragments were selected for determining their crystal structures [167]. Out of them, six formed steric zippers, including _300_GNNQGSN_306_, _321_AMMAAA_326_, _328_AALQSS_333_, _333_SWGMMGMLAS Q_343_, _370_GNNSYS_375_, and _396_GFNGGFG_402_, which demonstrated tight side chain interdigitation, similar to previously reported zippers in other amyloidogenic proteins, such as β -amyloid [168]. All six structures are composed of in-register sheets. While _321_AMMAAA_326_ and _333_SWGMMGMLASQ_343_ form antiparallel sheets, the other four form parallel sheets. On the other hand, four segments such as _312_NFGAFS_317_ form a kinked β-sheet structure like LARKS, which forms labile aggregates. Two familial variants within this segment, A315T and A315E, together with phosphorylation, appeared to strengthen the reference sequence assembly, making it irreversible by creating a more stable structure with stronger interaction between each of the sheets [167,168].

Furthermore, cryo-EM structures of two long segments of the pathogenic cores of human TDP-43 aggregation have been reported [169]: namely SegA (residues 311-360), which forms three polymorphs, all with dagger-shaped folds (I-III of Fig. 6I). On the other hand, SegB (residues 286-331 containing ALS-causing mutation A315E) forms R-shaped folds (IV of Fig. 6I). Energetic analysis suggests that the dagger-shaped polymorphs represent irreversible fibril structures, whereas the SegB polymorph may participate in both reversible and irreversible fibrils. These structures again highlight the polymorphic nature of amyloid fibrils formed by TDP-43 PLD, and showed how the A315E mutation converts the R-shaped polymorph to an irreversible form that enhances pathology.

The cryo-EM structure of amyloid has also been determined for the entire TDP-43 PLD (276-414) [170]. This structure reveals single protofilament fibrils containing a large (139-residue), tightly packed core (V of Fig. 6I). While the C-terminal part of this core region is largely planar and characterized by a small proportion of hydrophobic amino acids, the N-terminal region contains numerous hydrophobic residues and has a non-planar backbone conformation, resulting in rugged surfaces of fibril ends (V of Fig. 5I). The structural features found in these fibrils differ from those previously found for fibrils generated from short protein fragments.

Recently, the cry-EM structures have been determined for aggregated TDP-43 in the frontal and motor cortices of two individuals who had ALS with FTLD [171]. An identical amyloid-like filament structure comprising a single protofilament was found in both brain regions and individuals. The ordered filament core spanning residues 282-360 of TDP-43 PLD adopts a previously undescribed double-spiral-shaped fold (VI of Fig. 6I), which shows no similarity to those of TDP-43 filaments formed *in vitro*. An abundance of glycine and neutral polar residues facilitates numerous turns and restricts β-strand length, which results in an absence of β-sheet stacking that is associated with cross-β amyloid structure. An uneven distribution of residues gives rise to structurally and chemically distinct surfaces that face external densities and suggest possible ligand-binding sites. The nucleus of the double-spiral fold is formed by the hydrophobic region. Two hydrophobic clusters are located on either side of the main chain. The glycine-rich region towards the N terminus and the Q/N-rich region towards the C terminus form spiral branches that wrap around the hydrophobic nucleus and bury the remaining hydrophobic residues, with the exception of M359 at the C terminus. The ten β-strands within the fold (β1-β10) are short compared to those of amyloid structures, with only two (β6 in the hydrophobic nucleus and β9 in the Q/N-rich spiral branch) being longer than three residues. Stacking of TDP-43 molecules with this fold gives rise to parallel in-register inter-molecular β-sheets that extend along the helical axis, similar to amyloid structures. The inter-strand segments consist mainly of turns introduced by glycine residues and hydrogen bonds between buried polar side chains, main chain peptide groups and ordered solvent molecules. Between β4 and β5, there is a peculiar structural motif of three conjugated β-turns that was previously observed in the β-helix domain of glutamate synthase and related structures23. The brain-derived TDP-43 filaments are structurally different from filaments assembled in vitro from its LC domain or fragments thereof. They have opposite chirality and differ both in protein fold and secondary structure. The structure of TDP-43 filaments from ALS with FTLD establishes the structural characterization of aggregated TDP-43 from human brain. It revealed the formation of filaments that are structurally distinct from amyloid filaments in other neurodegenerative diseases. The conserved filament fold of pathological TDP-43 in ALS with FTLD guides the development of accurate disease models, as well as diagnostic and therapeutic agents.

## 4. Misfolding/aggregation-prone proteins disrupt the dynamics of LLPS.

Emerging experimental evidence indicates that proteins prone to misfolding and aggregation have a general ability to accumulate in phase separated droplets, disrupting their dynamics and fostering their solidification. For example, extensive observations unraveled that the accumulation of aggregation-prone proteins into SGs resulted in their abnormal dynamics, which has been proposed as a potential mechanism of gain of toxicity for aggregation-prone proteins to cause diseases and aging [37-39,172-174]. Nevertheless, the underlying biophysical basis remains largely unknown for the interactions between aggregation-prone proteins and phase separated assemblies.

**Figure 7.**
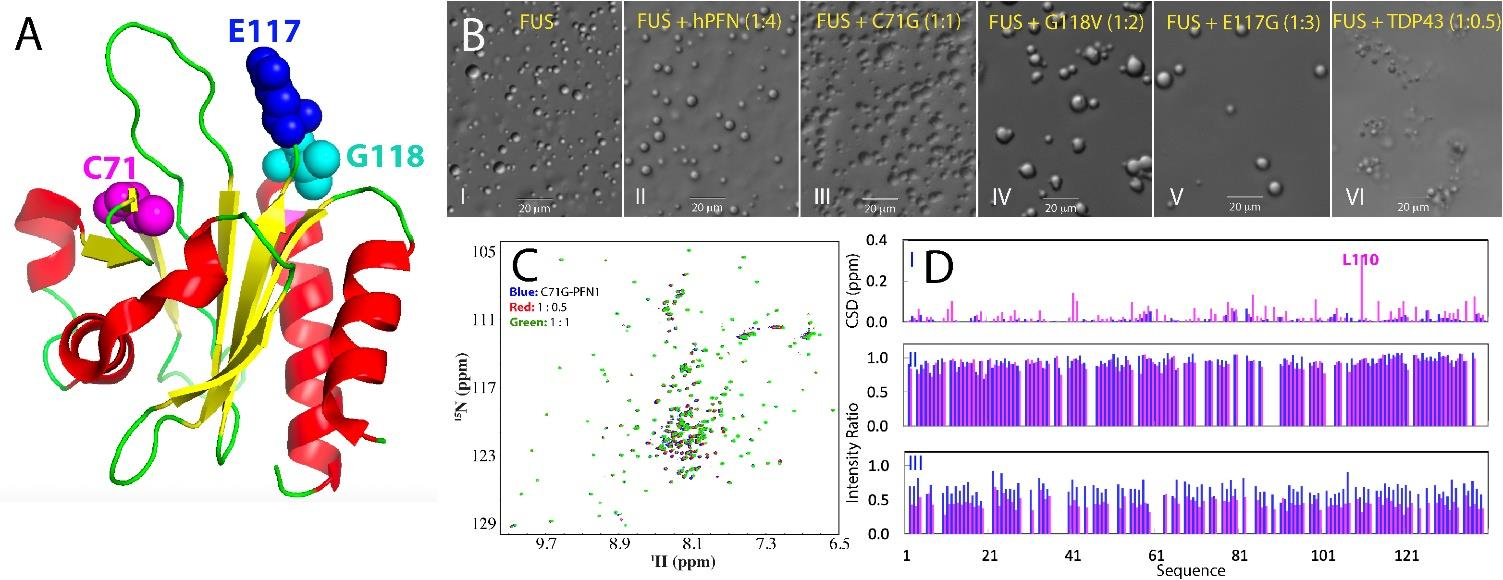
ALS-causing hPFN1 mutants differentially disrupt LLPS of FUS prion-like domain. (A) Structure of hPFN1 with residues Cys71, Glu117 and G118 displayed in spheres. (B) DIC microscopy images of liquid droplets of FUS PLD (I); and in the presence of WT-hPFN1 (II); C71G (III); G118V IV); E117G (V); TDP-43 (10-102) (VI). (C) Superimposition of HSQC spectra of ^15^N-labeled C71G-hPFN1 in the presence of unlabeled FUS PLD at different molar ratios. (D) (I) Chemical shift difference (CSD) of HSQC peaks of ^15^N-labeled C71G-hPFN1 in the absence and in the presence of unlabeled FUS PLD at molar ratio of 1:1 (C71G:FUS) for the folded (blue) and unfolded (purple) states. Normalized residue-specific ratios of HSQC peak intensity of the folded (II) and unfolded (III) states of C71G-hPFN1 in the presence of FUS PLD at 1:0.1 (blue) and 1:1 (purple).

Human profilin-1 (hPFN1) is a 140-residue protein that plays a crucial role in regulating actin polymerization and other cellular functions [175]. It possesses a globular fold consisting of a seven-stranded antiparallel β-sheet flanked by N- and C-terminal α-helices on one side, while three small helical segments are located on the opposite side of the sheet (Fig. 7A). Several mutations in hPFN1 including C71G, E117G, and G118V have been identified as causative factors in ALS. Among them, C71G-hPFN1 exhibits the highest proneness to aggregation and toxicity, leading to ALS phenotypes in mice through a gain of toxicity mechanism [29-32,176]. Interestingly, previous research has demonstrated that C71G-hPFN1 can induce seed-dependent co-aggregation with FUS/TDP-43, leading to prion-like propagation. However, the underlying mechanism of this phenomenon remains largely unexplored [42].

Very recently, an *in vitro* biophysical study focused on examining the impact of three ALS-causing hPFN1 mutants on the dynamics of phase-separated droplets of FUS NTD (1-267), followed by further high-resolution NMR characterization of the conformations and dynamics of both the hPFN1 mutants and FUS NTD [41]. As shown in I of Fig. 7B, FUS NTD could undergo phase separation, resulting in the formation of liquid droplets, with some reaching a diameter of approximately 10 μm. Despite their large size, these droplets maintain their dynamic nature and exhibit Brownian motion, as observed through DIC imaging [41]. The addition of WT-hPFN1 to the phase-separated FUS PLD sample does not elicit any large alteration in the dynamics of the liquid droplets even with a molar ratio of up to a molar ratio of 1:4 (II of Fig. 7B). In stark contrast, the addition of C71G-hPFN1 at only 1:0.5 noticeably decreased the dynamics of the droplets and seemed to induce their clustering and coalescence. When C71G-hPFN1 was added at 1:1, it completely suppressed the Brownian motion of the large droplets (III of Fig. 7B). Moreover, further addition of C71G-hPFN1 at 1:1.5 resulted in the formation of a gel-like state with significantly increased viscosity [41].

It is worth noting that the addition of G118V-hPFN1, which exhibits lower ALS-causing toxicity compared to C71G-hPFN1, at 1:1, resulted in only a slight increase in the coalescence of small droplets to merge into larger droplets. However, when G118V-hPFN1 was added at 1:2, it deformed the round shape of the droplets and disrupted their Brownian motion (IV of Fig. 7B). Furthermore, upon further addition to 1:3, G118V-hPFN1 also induced the formation of a gel-like state. When E117G-hPFN1, which exhibits the weakest ALS-causing toxicity, was added even at 1:3, it appeared to primarily enhance the coalescence of small droplets into larger ones without causing any significant alteration in the dynamics of the liquid droplets (V of Fig. 7B). It was only upon further addition at a ratio of 1:4 that the formation of a gel-like state was observed.

In order to investigate whether other aggregation-prone proteins have similar effects on liquid droplets of FUS NTD as observed with hPFN1 mutants, the impact was examined using TDP-43 (10-102), which is completely unfolded due to the deletion of the first 9 residues that form the initial β-strand (I of Fig. 4B). Remarkably, even at 1:0.1, the addition of TDP-43 (10-102) was sufficient to disrupt the large droplets to fragmentize into smaller ones. Notably, many droplets were connected in a "beads-in-a-string" arrangement. With further addition to 1:0.5, visible aggregates were also formed (VI of Fig. 7B), and at 1:1, the sample transitioned into a gel-like state mixed with aggregates.

To obtain detailed insights, a step-wise titration of ^15^N-labeled C71G-hPFN1 was conducted by adding unlabeled FUS PLD, and the process was monitored using NMR HSQC spectra (Fig. 7C). As previously characterized, C71G-hPFN1 exists in equilibrium between the folded and unfolded states [32,176]. Interestingly, as FUS PLD was gradually added, the chemical shifts (I of Fig. 7D) and intensities (II of Fig. 7D) of HSQC peaks corresponding to the folded state of C71G-hPFN1 remained largely unaffected. In contrast, the unfolded state of C71G-hPFN1 displayed significant shifts in almost all HSQC peaks (Fig. 7C), such as Leu110 with a chemical shift difference (CSD) of 0.33 ppm (I of Fig. 7D). Notably, these shifted HSQC peaks were distributed throughout the entire sequence of C71G-hPFN1, indicating that the shifts may be a result of weak but multivalent interactions between the unfolded state of C71G-hPFN1 and FUS PLD.

Likely, when C71G-hPFN1 is unfolded, its previously buried residues become exposed and accessible for interactions with the residues of FUS PLD lacking of any large hydrophobic residues. These interactions can be complex and may involve various mechanisms such as π-π interactions between aromatic residues, π-cation interactions between aromatic residues and Arg/Lys residues, as well as electrostatic and hydrophobic interactions. Furthermore, the accumulation of the unfolded state of C71G-hPFN1 within the liquid droplets of FUS PLD may induce self-association or/and dynamic aggregation of the unfolded state, which could contribute to the observed shifts in HSQC peaks. Consistent with the notion of weak and multivalent interactions, the intensity of most HSQC peaks was significantly reduced even at a ratio of 1:0.1, and at a ratio of 1:1, the intensity of most HSQC peaks decreased by an average of approximately 50% (III of Fig. 7D). This strongly indicates that the folded state of C71G-hPFN1 was not substantially affected in the presence of FUS PLD. In contrast, the unfolded state exhibited weak but multivalent interactions with FUS PLD and/or induced self-association. As a result, NMR peaks corresponding to most residues in the unfolded state of C71G-hPFN1 became significantly broadened, and their peak intensities were considerably reduced.

Strikingly, very recently a study was dedicated to understanding how the unfolded states of the intrinsically foldable proteins (IFPs) drive phase separation and the formation of unfolded protein deposits (UPODs) [177]. IFPs have been shown to exhibit a diverse spectrum of thermodynamic stabilities determined by sequences and fold types [178]. Disease-related proteins such as hPFN1 belong to the sub-proteome with the least thermally stable IFPs. These proteins can readily sample the unfolded states even under physiological conditions and are more vulnerable to mutations. Here, for example, the C71G mutation even destabilized hPFN1 into the co-existence of both folded and unfolded states [176]. This study showed that the unfolded states of these proteins may undergo aggregation-mediated phase separation which is driven by homotypic interactions predominantly involved in aromatic and large hydrophobic residues [177].

The findings from ALS-causing hPFN1 mutants further extend the conclusion [177], demonstrating that the unfolded state, rather than the folded state, of IFPs like C71G-hPFN1 can also interfere in phase separation of intrinsically disordered proteins like FUS NTD through heterotypic interactions. In the specific case of C71G-hPFN1 and FUS NTD, these heterotypic interactions may primarily involve aromatic and Arg/Lys residues due to the absence of large hydrophobic residues in FUS NTD. Moreover, co-phase separation of C71G-hPFN1 and FUS NTD is to significantly increase their local concentrations and enhance association, thus disrupting the dynamics of phase separation and leading to eventual aggregation.

**Figure 8.**
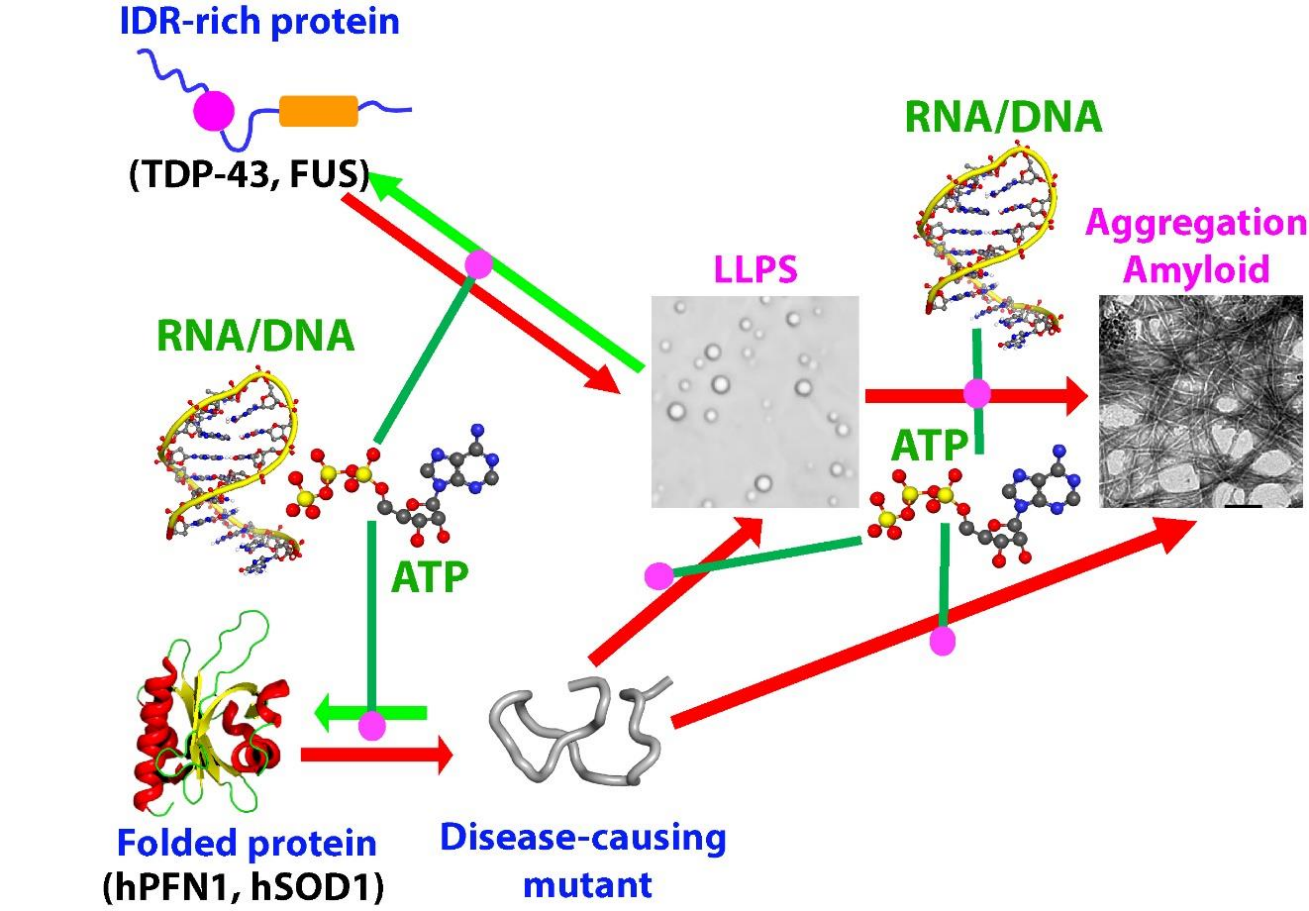
Interplay of IDR-rich proteins and mutants of folded proteins in LLPS and amyloid fibrillation, which is universally mediated by ATP and nucleic acids.

## 5. Summary, open questions and limitations

Eukaryotic cells house a variety of subcellular organelles, such as nuclei, mitochondria, lysozomes, peroxisomes, chloroplasts, and the endoplasmic reticulum/Golgi apparatus, each enclosed by specialized phospholipid membranes of integral importance to their functions [179]. On the other hand, while the presence of other cellular puncta lacking phospholipid bilayer membranes has been long recognized, it has only been recently established that these membrane-less organelles and condensates are commonly formed through LLPS. Consequently, the study of membrane-less organelles and condensates is still in its infancy and numerous fundamental questions remain unanswered.

This review focuses on LLPS, aggregation, and amyloid fibrillation of two ALS-causing protein categories: IDR-rich FUS and TDP-43, alongside ALS-causing mutants of the well-folded hPFN1. FUS and TDP-43 are composed of folded domains and IDRs, each contributing uniquely to their LLPS and amyloidosis. Briefly, FUS contains over 70% of IDRs, which can be grouped into two types: a Tyr-abundant prion-like domain and three RG-/RGG-rich regions. The isolated prion-like domain can phase separate independently only at high concentrations and readily transition into labile hydrogel and amyloid fibrils, whereas the RG-/RGG-rich regions are incapable of phase separation or undergoing amyloid fibrillation. However, LLPS of the FUS prion-like domain can be significantly enhanced to occur at low concentrations either by covalently linking it to RGG1 or by simply adding the isolated RGG3. This implies that in the full-length FUS, LLPS may occur with different pathways driven by distinct forces. Notably, ATP, RNA and DNA all can biphasically modulate LLPS of FUS. Most importantly, despite having much lower binding affinity than RNA and DNA, ATP can bivalently bind to Arg/Lys residues like RNA and DNA. Consequently, it has the ability to displace RNA and DNA from their binding sites on FUS, leading to the dissolution of nucleic-acid-induced LLPS of FUS. Intriguingly, FUS RRM appears to have relatively low thermodynamic and kinetic stability, making it vulnerable to amyloidosis. However, ATP can bind to the specific pocket on FUS RRM, kinetcally inhibiting the amyloidosis of FUS RRM.

TDP-43 comprises a unique N-terminal domain (NTD), two RRM domains, and a C-terminal prion-like domain (PLD). This PLD contains an evolutionarily-conserved hydrophobic region that interacts with membranes and undergoes a pH-dependent alpha-to-beta conformational transformation essential for LLPS. Importantly, TDP-43 exhibits dynamic inter-domain interactions, thus likely adopting the open and closed states exchanging on μs-ms time scale. TDP-43 appears to have two distinct pathways of LLPS: one is mediated by NTD-oligomerization, and the other is PLD-mediated through the alpha-to-beta conformational transition. Recent *in vivo* studies have revealed that these two distinctive LLPS pathways lead to differential functional and pathological consequences. Notably, two TDP-43 RRM domains also have relatively low thermodynamic stability, thus susceptible to amyloidosis. ATP also can bind to specific pockets on RRM1 and RRM2, effectively inhibiting their amyloidosis. Again, ATP and nucleic acids can biphascially modulate LLPS of FUS by binding to the specific sites on the folded domains including NTD and RRMs as well as Arg/Lys residues within IDRs.

LLPS appears to emerge as a crucial convergence for IDR-rich and misfolded proteins to initiate human diseases (Fig. 8). While well-folded globular proteins like hSOD1 and hPFN1 only undergo phase separation at exceptionally high concentrations, the disease-causing mutations render them to be either misfolded or completely disordered, thus gaining the characteristics of IDR-rich proteins [19,28,32,42,119,180,181]. These mutants may become capable of undergoing homotypic phase separation [177], or/and act to disrupt the dynamics of phase separation of IDR-rich proteins [32], thereby promoting the co-aggregation and amyloid fibrillation. Most strikingly, ATP appears to be capable of influencing all these processes through specific binding or/and nonspecific salt/electrostatic effects in the context-dependent manner (Fig. 8). In particular, ATP has been recently decoded to energy-independently induce folding of ALS-causing C71G-hPFN1 and hSOD1 by mediating protein hydration at the highest efficiency known so far [100,176].

Nevertheless, many important questions still remain to be addressed, including but not limited to: 1) Why do the folded domains of FUS and TDP-43 have relatively low thermodynamic stability and high dynamics. Is this feature needed for their functions? 2) Both FUS and TDP-43 have multiple domains or regions that can independently undergo phase separation. So, what is the interplay among these independent capacities in LLPS? How does the interplay modulate the overall LLPS behaviors? Whether does FUS also have distinct pathways of phase separation for different functions, such as found for TDP-43? 3) What is the relationship between their LLPS in the absence and presence of nucleic acids in the context of their functions? 4) What is the interplay of ATP's roles, as a general competitor with nucleic acids for LLPS, and its specific role, such as enhancing NTD-mediated LLPS of TDP-43? 5) Is the transition of LLPS into aggregation or amyloid fibrillation an unavoidable exaggeration, or does it serve specific functional roles? 6) Does the interference of the unfolded states of disease-associated mutants of folded proteins in LLPS of IDRs represent a general mechanism for misfolding/aggregation-prone proteins to gain toxicity?

One central goal of biophysical studies is to ultimately facilitate design of drugs targeting LLPS and amyloidosis, which could mediate protein homeostasis within cells and lead to new treatments for currently intractable diseases, particularly neurodegenerative diseases such as ALS, FTD and aging. For example, current therapeutic approaches for neurodegenerative diseases often focus on alleviating symptoms rather than addressing the underlying causes. In this context, understanding the molecular mechanisms governing phase separation can aid in identifying targets for disrupting harmful condensate formation and restoring normal cellular function. Elucidating the factors that promote aggregation and amyloid formation can facilitate the development of strategies to prevent or disaggregate these toxic protein aggregates. In this context, ATP, a universal regulator of protein folding, stability, dynamics, interactions, LLPS, and aggregation/fibrillation, thus represents a key starting point for design of small molecule drugs to treat human disease and aging.

On the other hand, *in vitro* studies have several major limitations: 1) they typically use purified proteins under controlled conditions, which does not accurately reflect the complex cellular environment. Within cells, the dynamic environments involve a complex interplay of factors like pH, temperature, and ionic strength, all capable of influencing the behavior and even structures of proteins. Indeed, an elegant example is the hydrophobic region of TDP-43 PLD, which can adopt pH-dependent secondary structures with different functional roles. Moreover, cellular components like lipids, RNA, and proteins interact with one another, influencing and modifying their respective behaviors. 2) *In vitro* studies often do not take into account the spatial organization of cells. However, the specific locations of phase-separated condensates or amyloid aggregates might determine their functionalities. For instance, methionine oxidation within the membrane-interacting helix of TDP-43 PLD appears to alter the functional role of TDP-43 by relocalizing it onto mitochondria [135]. 3) *In vitro* studies fail to recapitulate the dynamic nature of cells, where constant changes can significantly impact protein behaviors. For example, changes in gene expression can alter the levels of proteins. Therefore, it is crucial to complement *in vitro* studies with *in vivo* investigations to gain a comprehensive understanding within the context of complex cellular environments. Ultimately, a challenging yet pivotal direction lies in developing novel techniques to visualize LLPS, aggregation, and amyloid fibrillation within living cells [182,183].
